# Safety of Daily Co-Trimoxazole in Pregnancy in an Area of Changing Malaria Epidemiology: A Phase 3b Randomized Controlled Clinical Trial

**DOI:** 10.1371/journal.pone.0096017

**Published:** 2014-05-15

**Authors:** Christine Manyando, Eric M. Njunju, David Mwakazanga, Gershom Chongwe, Rhoda Mkandawire, Davies Champo, Modest Mulenga, Maaike De Crop, Yves Claeys, Raffaella M. Ravinetto, Chantal van Overmeir, Umberto D’ Alessandro, Jean-Pierre Van geertruyden

**Affiliations:** 1 Tropical Diseases Research Centre, Ndola, Zambia; 2 Choma District Health Management Team, Choma, Zambia; 3 Institute of Tropical Medicine, Antwerp, Belgium; 4 Medical Research Council, Fajara, The Gambia; 5 International Health Unit, University of Antwerp, Antwerp, Belgium; 6 Department of Pharmaceutical and Pharmacological Sciences, KU Leuven, Leuven, Belgium; Kenya Medical Research Institute (KEMRI), Kenya

## Abstract

**Introduction:**

Antibiotic therapy during pregnancy may be beneficial and impacts positively on the reduction of adverse pregnancy outcomes. No studies have been done so far on the effects of daily Co-trimoxazole (CTX) prophylaxis on birth outcomes. A phase 3b randomized trial was conducted to establish that daily CTX in pregnancy is not inferior to SP intermittent preventive treatment (IPT) in reducing placental malaria; preventing peripheral parasitaemia; preventing perinatal mortality and also improving birth weight. To establish its safety on the offspring by measuring the gestational age and birth weight at delivery, and compare the safety and efficacy profile of CTX to that of SP.

**Methods:**

Pregnant women (HIV infected and uninfected) attending antenatal clinic were randomized to receive either daily CTX or sulfadoxine-pyrimethamine as per routine IPT. Safety was assessed using standard and pregnancy specific measurements. Women were followed up monthly until delivery and then with their offspring up to six weeks after delivery.

**Results:**

Data from 346 pregnant women (CTX = 190; SP = 156) and 311 newborns (CTX = 166 and SP = 145) showed that preterm deliveries (CTX 3.6%; SP 3.0%); still births (CTX 3.0%; SP 2.1%), neonatal deaths (CTX 0%; SP 1.4%), and spontaneous abortions (CTX 0.6%; SP 0%) were similar between study arms. The low birth weight rates were 9% for CTX and 13% for SP. There were no birth defects reported. Both drug exposure groups had full term deliveries with similar birth weights (mean of 3.1 Kg). The incidence and severity of AEs in the two groups were comparable.

**Conclusion:**

Exposure to daily CTX in pregnancy may not be associated with particular safety risks in terms of birth outcomes such as preterm deliveries, still births, neonatal deaths and spontaneous abortions compared to SP. However, more data are required on CTX use in pregnant women both among HIV infected and un-infected individuals.

**Trial Registration:**

Clinicaltrials.gov NCT00711906.

## Introduction

Malaria is one of the most important causes of morbidity and mortality worldwide, with children and pregnant women being the most severely affected groups [1]. In low transmission areas, all pregnant women have little or no pre-existing immunity and malaria can evolve towards severe disease with a higher risk of maternal and perinatal mortality. Foetal and perinatal loss can be as high as 60–70% [2]–[6]. In high transmission areas, primigravidae are more at risk than multigravidae and malaria infection is associated with maternal anaemia, low birth weight (LBW) and stillbirth [7], [8]. Annually, malaria during pregnancy is estimated to account for 5% of the cases of severe anaemia in pregnant women, approximately 35% of preventable low birth weight, 3–8% of infant mortality and 5,000 to 200,000 infant deaths [9].

Anaemia, malnutrition and HIV infection are also common events in malaria endemic areas and contribute to LBW. HIV infection in pregnancy is associated with an increased risk of malaria infection and higher parasite densities [10]–[12]. The risk increment is more pronounced in multigravidae than in primigravidae, indicating that HIV-1 hinders the development of immunity [10], [13], [14]. HIV-1 infection is also associated with lower birth weight [15], higher infant mortality and a 4-fold greater risk of malaria attack in the new-born [16]. The increased risk of placental malaria in HIV infected mothers is also associated with a higher post-natal mortality [17]. The risk of anaemia suggests a synergistic interaction between HIV and malaria, placing dually infected women at very high risk of developing severe anaemia [18], [19].

The World Health Organization (WHO) recommends a package of interventions for the prevention and control of malaria in pregnancy. This comprises intermittent preventive treatment (IPTp), use of insecticide treated materials (ITMs) [20], [21], and rapid access to effective case management for malaria illness and anaemia [22]. Prevention with efficacious antimalarials can reduce the incidence of placental malaria, LBW and maternal anaemia [23], [24]. IPTp with sulfadoxine/pyrimethamine (IPTp-SP) has been proven to be an effective strategy to reduce the burden of malaria during pregnancy. It is based on administering at least 2 treatment doses of SP to pregnant women after quickening (around 18–20 weeks) at the Antenatal Care (ANC) centres [25].The dose of SP should be given at not less than one month interval as stipulated in the updated WHO IPTp guidelines, which recommends IPTp at every scheduled antenatal visit [26]. HIV-1 infection may decrease the efficacy of IPTp-SP but 2 or more doses of SP in the second and third trimester still reduce peripheral and placental malaria, and maternal anaemia, including severe anaemia, and increase birth weights [27], [28]. Unfortunately, the beneficial effect of IPTp-SP is currently threatened by increasing resistance to SP. Therefore, other antimalarials to be used as IPTp are currently investigated.

Cotrimoxazole (CTX) has been used for treating malaria in children; recently, its daily use by non-pregnant HIV-infected adults was associated with 70% reduction in the incidence of clinical malaria [18], [29], [30]. Antibiotic therapy during pregnancy may be beneficial and reduce some adverse pregnancy outcomes [31]. CTX prophylaxis significantly improves birth outcomes in HIV infected women with <200 CD4 cells/µl with reduction of chorionamnionitis, prematurity and neonatal mortality [32]. A study conducted in Zambia using historical controls concluded that antenatal provision of CTX was beneficial for HIV-infected pregnant women with low CD4 count but not in women with ≥200 CD4 cells/µl [32]. However, it is important to note that this study was conducted in an area of very low malaria risk; CTX may have had a different impact if malaria transmission had been substantial. Though daily CTX could be considered as a potential alternative for IPTp-SP, no information on its effectiveness in preventing malaria infection during pregnancy and its consequences (maternal anaemia and LBW) is available [33].

A clinical trial comparing the protective efficacy of daily CTX versus IPTp-SP in pregnant women was carried out in southern Zambia. Its primary objective was to test the hypothesis that CTX prophylaxis is not inferior to SP prophylaxis in reducing placental malaria. The secondary objectives were to evaluate efficacy of CTX prophylaxis in preventing malaria peripheral parasitaemia, perinatal mortality and in improving birth weight; to establish the safety on the offspring by measuring the gestational age and birth weight at delivery, and also to compare the safety and efficacy of CTX prophylaxis to that of SP based on these parameters. The study participants were stratified by HIV status in order to follow the national preventive guidelines, taking note of the fact that pregnant women with CD4 count <200 cells/µl (at the time of the trial as this has now been revised to <350 cells/µl) are given daily CTX and should not receive SP-IPTp. However, at the time of implementation and thanks to the coordinated efforts for malaria control, malaria transmission had become extremely low to the extent that the study was prematurely stopped because it would not have had sufficient power to meet the original objectives. However, before interrupting the study, several pregnant women were included and received either daily CTX or IPTp-SP in a 1∶1 ratio. This provided the opportunity to report the safety of daily CTX in an area of low malaria endemicity.

## Methods

### Study Area

Choma district is located in the Southern Province of Zambia. Though malaria continued to be a key focus of public health services in Choma as is the case in Zambia, there has been large scale up of antimalarial interventions and considerable progress in control of disease in recent years [22], [34]. The malaria burden decreased, in a population of 12 million, from 3.3 million reported cases and 9,369 deaths in 2003 to 2.9 million cases and 3,862 deaths in 2009 [22].

The changing epidemiology was documented in Livingstone, less that 200 km from Choma, where the number of malaria cases declined from 8000 per quarter between 2004 and 2007[35] to only 65 cases in the third quarter of 2008. Reported malaria deaths declined from 60 in 2004 to zero in 2009 [35]. As Choma and Livingstone are in the same epidemiological zone, these reports retrospectively confirm that in 2009 the malaria risk in Choma had decreased to an extremely low level and support the decision of interrupting the clinical trial prematurely.

### Decision to Stop the Trial in Choma

The study was initiated in Choma area in January, 2009, shortly after a large observational study that was recruiting pregnant women with history of clinical malaria and receiving antimalarial treatment had just been concluded. The screening for the trial (the Malcotrim study) included peripheral blood smear analysis. After 6 months of patient recruitment a review of the data on the screening smear results revealed that only 4 of the 421 screened patients had a malaria infection. This prompted the study director in liaison with entire investigational team to inform the ethical review committee at the Tropical Diseases Research Centre (TDRC) and the National Ethical Review Committee, the sponsor as well as the Zambia Medicines Regulatory Authority of the extremely low malaria prevalence in the study area. As the risk of malaria infection in pregnant women was much lower than expected, the trial would not have had sufficient power to provide the required answer, i.e. non-inferiority of the CTX arm in preventing placental malaria as compared to SP intermittent preventive treatment. The issue of changing the study site was discussed also with the DSMB that received and reviewed on a quarterly basis (unless differently requested by the DSMB members) the updates on screening and recruitment and on losses to follow-up, as well as the quarterly SAE reports routinely sent also to the Ethical Committees. In addition, Serious Adverse Events were also reported on a case-by-case basis, unless differently requested by the DSMB members, in order to allow them to promptly comment on specific events. The decision to move the study site to another location known to have high malaria transmission was taken by the research team in consultation with the DSMB, ethics committees, the Regulatory authority and the sponsor.

### Study Participants

Pregnant women between 16 and 28 weeks of gestation attending the antenatal clinic of the Shampande Health Centre or Choma Hospital in Choma District, accepting to be tested for HIV and willing to participate in the study were enrolled. The inclusion criteria were absence of symptoms consistent with malaria at the time of recruitment (determined by asking them; a positive blood smear was not an exclusion criterion although all women found with a malaria infection at the time of recruitment were also asked if they had any symptoms of malaria), willingness to deliver at the health facility and willingness to adhere to study requirements, e.g. monthly visits to the antenatal clinic. Exclusion criteria were history of allergy to sulpha drugs, history or presence of major illnesses likely to influence pregnancy outcome such as diabetes, severe renal or heart disease or active tuberculosis; intent to move outside the study catchment area before delivery, severe anaemia (Hb<7 g/dl) and previous history of unfavourable pregnancy outcome (such as but not limited to pre-eclampsia, caesarean section, stillbirth). Recruitment was conducted from February to September, 2009.

### Ethical Considerations

The study was sponsored by the Institute of Tropical Medicine, Antwerp. It was approved by the Institutional Review Board at the Institute of Tropical Medicine, Antwerp, and the Ethics committees at the University of Antwerp and at the Tropical Diseases Research Centre, Ndola, Zambia. These bodies also approved the Amendment 1.0 of 25 March 2009, which was issued to align the study to the new management practices for prevention of mother to child transmission (PMTCT) in Zambia (pregnant women to be treated with anti-retroviral therapy (ART) if their CD4 count was <350 cells/µl). All patients were informed about the study before they were asked to sign the informed consent. Each study participant was assigned a unique patient code that was used for all subsequent forms in the study, so that confidentiality of all subjects was guaranteed. A no fault study insurance was taken to indemnify possible damage linked to a subjects participation in the study. The trial was registered in the Clinicaltrials.gov registry, identifier: NCT00711906, URL: http://clinicaltrial.gov/ct2/show/NCT00711906.

### Study Design

This was a phase 3 randomized open label clinical trial whose planned primary endpoint was placental malaria. (The protocol for this trial and supporting CONSORT checklist are available as supporting information; see CONSORT Checklist S1 and Protocol S1). The sample size calculation for the HIV negative population was determined by assuming the prevalence of placenta malaria at 10% in both groups with assumptions that CTX prophylaxis was considered equivalent to SP if the two-sided 95% confidence interval was below 15% (90% power was required for the primary hypothesis). Therefore, 788 pregnant women per treatment arm were required with an assumed loss to follow up of 10%. For HIV positive women with CD4 count ≥200 cells/µl, similar assumptions were considered though the two-sided 95% confidence interval was expected to be below 20%. Therefore, 260 pregnant women per treatment arm were required with an assumed loss to follow up of 10%. Therefore, total sample size initially planned for the study was 2,096 pregnant women.

**Figure 1:**
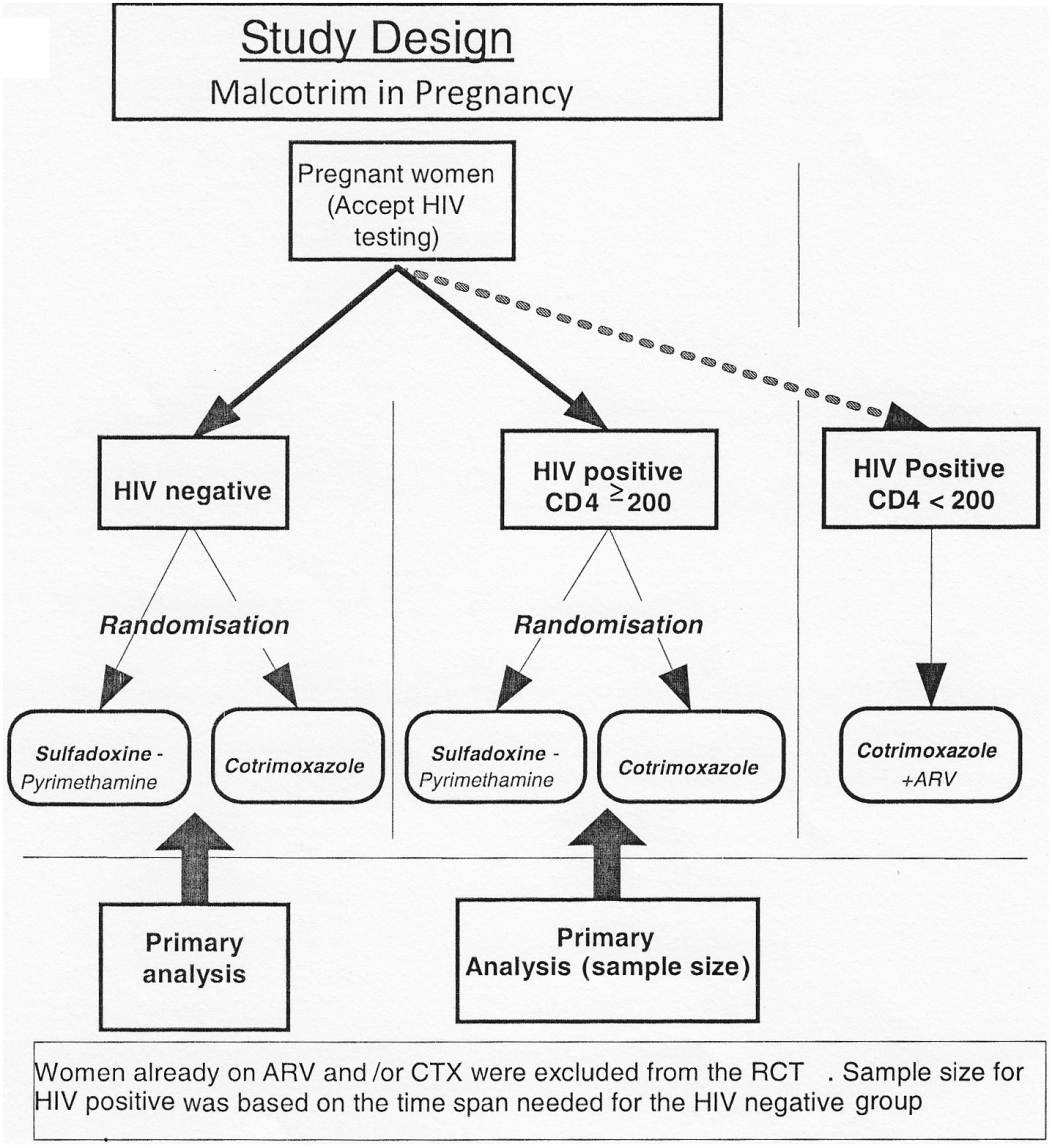
Study Design.

The study stratification by HIV status to correspond to the study hypothesis and objectives (as the study aimed at describing differences in pregnancy outcomes by HIV status) is shown in figure 1.

All HIV negative and HIV positive with CD4 count ≥200 cells/µl were randomized to either three monthly doses of SP–IPT (a product of Roche, Fansidar) or daily CTX prophylaxis (2 tablets of 400 mg of sulphamethoxazole and 80 mg of trimethoprim; a product of Roche, Bactrim). Therefore, the study intervention and randomization did not vary by HIV status in these two groups of women. Nevertheless, HIV positive pregnant women with CD4 count <200 cells/µl and complying with all other entry criteria were recruited in an observational cohort as they had to be given 2 tablets of CTX prophylaxis daily and antiretroviral treatment as per standard guidelines. In Zambia CTX prophylaxis for these women is part of the routine ANC care since November, 2003, in accordance with the WHO policy [37]. Thus, those women already on CTX prophylaxis and/or ARV treatment and complying with all other entry criteria were also recruited in this observational cohort.

At enrolment, a standardized questionnaire to collect demographic information, history of malaria episodes, past medical and obstetric history was administered. Other risk factors (such as smoking and drinking) that may affect pregnancy outcome were directly recorded in the Case Record Form (CRF). The socioeconomic status was also assessed on the basis of some key household parameters and a physical examination was performed. Laboratory tests at baseline included thick and thin blood smears for the diagnosis of malaria infection and measurement of haemoglobin. IPTp-SP administration was directly observed while CTX intake was supervised only for the first dose, the rest being taken at home until the next monthly visit when participants were reviewed.

### Randomization

The randomization was stratified by HIV status. Eligible women were randomized to one of the two arms according to a pre-defined randomization list prepared at ITM, Antwerp. Participants were assigned sequential study numbers which were matched with numbered envelopes containing the arm allocation that were opened by the study nurses only after recruitment of the study subject. There was no blinding as each of the study drugs was openly administered.

### Study Procedures

Women attended the antenatal clinic monthly for assessment of efficacy and safety parameters. Safety parameters included adverse events (AEs), serious adverse events (SAEs) and concomitant medications. During the monthly visits, physical examination, including foetal viability, was performed, information on bed net use was recorded, the study drugs provided, and compliance assessed by means of drug accountability. A blood sample for haemoglobin measurement (only once early in the third trimester, between 30–34 weeks), blood film for malaria parasites and a filter paper for later molecular biological assessments were collected (for efficacy assessment). Urine was analysed for glucose, proteins and blood using a dipstick urine test. At delivery, similar information as that collected at monthly visits was collected, including any AEs, SAEs and use of concomitant medication. In addition, a placenta blood sample for a thick blood film and later molecular analysis (filter paper) was collected from the maternal surface of the placenta. A biopsy was also obtained.

Women and their babies were seen at one and six weeks post-delivery. Pregnancy specific assessments included rates of stillbirth (>28 weeks gestation), neonatal mortality (≤28 days of birth), maternal mortality (up to 6 weeks post delivery), spontaneous abortion (≤28 weeks gestation), stillbirth, preterm delivery (≤37 completed weeks), incidence of low birth weight, gestational age at delivery (estimated from the last menstrual period [LMP], or by a developmental score [36], if the LMP was unknown), incidence of major and minor birth defects. Any infections that the patients reported (or were treated for) were also recorded.

### Data Management and Quality Control

Data were initially collected in a standardized source document template, and then entered into an electronic case report form (eCRF) using Macro 3.0 (InferMed Ltd, United Kingdom), a CFR21Part11 compliant software with an in built audit trail, password control and electronic signature. Each individual involved in this process (the data manager, data entry clerk, investigator and monitor) had a unique password-protected database user profile.

The eCRF comprised a data entry interface which was an exact but electronic copy of the source document (SD) and incorporated pre-programmed checks and warnings for inconsistencies, omission and commission errors. Data were entered with single data entry and then verified by the investigators who confirmed it with an electronic signature. A study monitor performed several external monitoring visits throughout the study, during which partial source data verification (SDV) was done. In case of inconsistencies, queries were raised in the eCRF. All findings were discussed with the entire study team at the end of each visit, so that corrective actions could be taken, and similar errors could be prevented in the future. Similarly, the investigators based at TDRC also performed SDV to resolve inconsistencies and answer queries raised by monitors and TDRC based data managers along with the study team on a monthly basis. In addition, a manual review of the database was done on key variables such as safety- and endpoint data. The system was set up on offline laptops and had a facility for uploading and saving data at a central server at the ITM. The data were extracted into SAS 9.2 (SAS Institute Inc., Cary, NC, USA) for formatting and statistical analyses.

### Statistical Analyses

Statistical analyses were based on the intention-to-treat principle (ITT). For continuous variables, tests of the normality of their distributions were done. Descriptive statistics means, medians, modes and standard deviations were determined as appropriate. Frequency and percentage distributions were computed for discrete variables. Additionally, asymptotic 95% confidence intervals (95% CI) for means of continuous variables were determined. Clopper-Pearson exact 95% confidence intervals were determined for percentage distributions of count variables. Missing data were assumed ignorable and thus all statistical analyses included only complete cases. Maternal and foetal outcomes such as still births and neonatal deaths; spontaneous abortions (≤28 weeks gestation); preterm deliveries (<37 completed weeks); neonatal mortality (within 28 days after birth) and birth weight, including LBW (<2,500 grams), were outcomes of interest.

For each of these variables, the treatment arms were analyzed per stratum (i.e. HIV status).

## Results

### The Trial Profile

**Figure 2:**
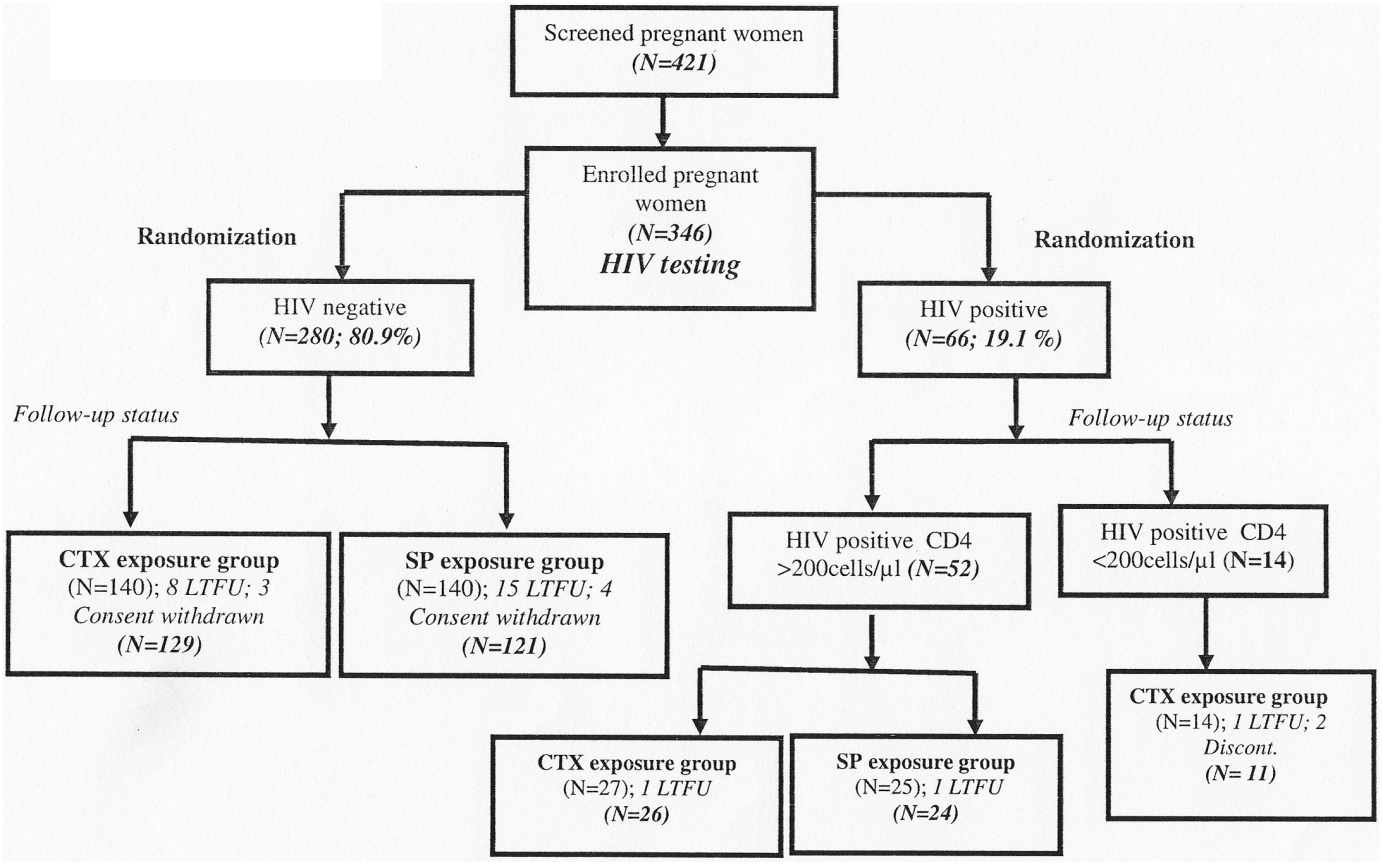
Trial Profile.

Four hundred and twenty one pregnant women were screened, 346 met the entry criteria and one additional patient was recruited among those whose baseline visit was at delivery (Figure 2). Gestational age ≤16 weeks or ≥28 weeks was the most frequent reason for non-enrolment in the study. Two hundred and eighty (280) HIV negative women and 52 HIV positive women with CD4 count ≥200/µl were randomized to either CTX (140 and 27 respectively) or SP (140 and 25 respectively). The 14 HIV positive pregnant women with CD4 count <200/µl continued on daily CTX according to the National guidelines for prevention of opportunistic infections in HIV infected pregnant women [37]. Due to loss to follow up and consent withdrawal (Figure 2), 166 pregnant women in the CTX arm and 145 in SP arm were analyzed at delivery.

### Demographic and Clinical Characteristics

Baseline characteristics including age, weight, height and gestational age were similar between treatment arms (Table 1). Most of women were married (although about a fifth of them declared to be single) and literate (Table 1). There was no difference in mean blood pressure and haemoglobin at baseline between study arms (Table 1).

**Table 1:** Baseline, clinical and socio-economic characteristics of all patients.

	Exposure groups^a^					
	Cotrimoxazole (CTX			Sulphadoxine-pyrimethamine (SP)		
Baseline and clinical Characteristics	N^b^ †	Mean	Standard deviation	N^b^ †	Mean	Standard deviation
Age	181	24.8	5.6	165	25.1	5.6
Weight (Kg)	181	60.2	10.4	165	60.2	10.3
Height (M)	181	159.5	11.3	163	159.8	5.7
Gestational age (wks)	181	18.2.	3.0	163	19.3	4.0
Systolic pressure	181	108.7	11.1	165	106.9	10.4
Diastolic pressure	181	68.8	9.7	165	68.0	9.1
Haemoglobin (g/dl)	181	12.1	1.3	165	12.0	1.4
Gravidity	180	2.0	2.0	164	2.0	2.0
**Socio-economic Characteristics**	**N** b	**%**	**95% CI**	**N** b	**%**	**95% CI**
Married	154	81.5	75.2, 86.8	114	73.1	64.9, 79.4
Single	34	18.0	12.8, 24.2	41	26.3	19.4, 43.7
Able to read	176	93.1	88.5, 96.3	143	91.7	85.5, 95.0
Able to write	180	95.2	91.2, 97.8	149	95.5	90.2, 97.8
That own house of residence	73	38.6	31.7, 46.0	61	39.1	31.2, 47.0
That rent house of residence	115	60.8	53.5, 67.8	94	60.3	51.8, 67.6
Using electricity for power	70	37.0	30.1, 44.4	51	32.7	25.2, 40.4
Using wood and charcoal for power	115	60.8	53.5, 67.8	103	66.0	57.6, 73.0
Using communal taps and pumps for water	121	64.0	56.7, 70.9	104	66.7	58.3, 73.6
Using taps inside house for water	46	24.3	18.4, 31.1	44	28.2	21.2, 35.7
Using wells for water	19	10.1	6.2, 15.3	7	4.5	1.8, 9.0

a *Exposure groups represent the treatment given for prevention of malaria; Kg = Kilograms; M = meters; wks = weeks.*

b *Enrolled pregnant women who gave informed consent,*

† *Numbers varying due to missing values.*

### Pregnancy Outcomes

Most pregnancy outcomes were spontaneous vaginal deliveries at gestational age of ≥37 weeks, with over 89% of the total deliveries between the 2 arms (Table 2). There were 10 preterm deliveries, 6 (3.6%) in the CTX and 4 (2.8%) in the SP arm, and few adverse pregnancy outcomes and neonatal deaths, with no difference between the study arms (table 2). Among the 10 caesarean sections, half of them were due to cephalo-pelvic disproportion (CPD). The rest were due to transverse lie (1), foetal distress (1), breech presentation (1) and pre-eclampsia (2).

**Table 2:** Overall Pregnancy outcomes by exposure group.

Characteristic	Overall population				HIV negative population				HIV positive population (CD4≥200cell/ µl)					HIV positive population (CD4<200cell/ µl)				
	CTX (N = 166)		SP (N = 145)		CTX (N = 129)		SP (N = 121)		CTX (N = 26)		SP (N = 24)		CTX (N = 11)			SP (Not Applicable-(N/A))		
Pregnancy outcomes	n	%	n	%	n	%	n	%	n	%	n	%	N		%	*-*	*-*	
Full-term normal deliveries*	150	90.4	130	89.6	118	91.5	108	89.2	25	96.2	22	91.7	7		63.6	-	-	
Preterm deliveries	6	3.6	4	2.8	5	3.9	4	3.3	0	0	0	0	1		9.1	-	-	
Spontaneous abortions	1	0.6	0	0	1	0.8	0	0	0	0	0	0	0		0	-	-	
Stillbirths	5	3.0	3	2.1	2	1.6	1	0.8	1	3.8	2	8.3	2		18.2	-	-	
Caesarean sections	4	2.4	6	4.1	3	2.3	6	5.0	0	0	0	0	1		9.1	-	-	
Neonatal deaths	0	0	2	1.4	0	0	2	1.7	0	0	0	0	0		0	-	-	
	**CTX (N = 161** § **)**		**SP (N = 142** ** **)**		**CTX (N = 127** ** **)**		**SP (N = 120** ** **)**		**CTX (N = 25** ** **)**		**SP (N = 22** ** **)**		**CTX (N = 9)**			**SP (N/A)**		
**Infant outcome**	**n**	**%**	**n**	**%**	**n**	**%**	**n**	**%**	**n**	**%**	**n**	**%**	**n**		**%**	**-**	**-**	
Low birth-weight	15	9.3	9	6.3	12	9.4	7	5.8	2	8.0	2	9.1	1		9.1	-	-	
Normal infant	146	90.7	133	93.7	115	90.6	113	94.2	23	92.0	20	90.9	8		90.9	-	-	

§ *Live births including one set of twins;*

**Live births only;

*Full-term normal deliveries = spontaneous vaginal deliveries at gestational age ≥37 completed weeks; N/A-Not applicable.

No congenital malformation was detected. There were 24 (8%) LBW babies, 15 (9.3%) in the CTX group and 9 (6.3%) in SP group (table 2). The mean birth-weight did not differ between study arms (3.1 kg; SD = 0.5) (Table 3). Pregnancy outcomes, including mean birth weight, did not differ by HIV status nor in HIV infected women by CD4 count (<200 cells/µl) (Tables 2 and 3). It is important to note that subgroup analyses were intended to be purely descriptive and were not powered for between-groups comparisons.

**Table 3:** Birth-weight profiles across the exposure groups.

	CTX		SP		Total	
Birth weight in kilograms	n**	Mean (SD)	n**	Mean (SD)	n**	Mean (SD)
Overall cohort	161	3.1 (±0.5)	142	3.1 (±0.5)	303	3.1 (±0.5)
HIV negative population	127	3.1 (±0.5)	120	3.2 (±0.5)	247	3.1 (±0.5)
HIV positive CD4>200 cells/µl	25	3.1 (±0.5)	22	3.2 (±0.5)	47	3.1 (±0.5)
HIV positive CD4<200 cells/µl	9	2.9 (±0.5)	-	-	9	2.9 (±0.5)

**Live births only;

*SD = Standard deviation.*

### General Safety Outcomes

A total of 61 AEs were observed in the 346 enrolled patients and there was no difference between study arms (Table 4). Most AEs were mild, i.e. 19 of the 61 (31.2%, with 9 from CTX arm and 10 from SP arm), and moderate 24 (39.3%) (12 AEs on CTX arm and 12 from the SP arm). Almost all these were either definitely unrelated or unlikely related to the study drug. Those reported to be severe and life threatening were 11 (18.0%) and 7 (11.5%), respectively and almost all were reported as SAEs (Table 4).

**Table 4:** Common adverse events by exposure group.

ADVERSE EVENTS AND SERIOUS ADVERSE EVENTS		Exposure group		
		CTX	SP	Total
**1.0 Adverse events reporting by MedDRA Primary System Organ Class: preferred term**				
**1.1 Infections and infestations** §				
Malaria		2	1	3
Respiratory Tract infection		1	1	2
Urinary Tract Infection		2	0	2
Vaginal candidosis		2	0	2
PROM/Threatened abortion		1	1	2
**1.2 Nervous system and other disorders** §				
Headache		2	0	2
General body pains		2	0	2
Gastro-intestinal disorders		1	3	4
Hypertension		3	2	5
General (Weakness, Fever, hypotension)		2	2	4
**2.0 Serious adverse events reported and their causal relationship to study drug**				
**Serious adverse Event**	**Causal relationship**	**CTX**	**SP**	**Total**
2.1. Preterm deliveries	*U*	4	3	9
	*D*	0	0	
	*P*	2	0	
2.1. IUFD and stillbirth	Unlikely	2	2	8
	Definitely unlikely	1	1	
	possible	2	0	
2.3. Caesarean section	*U*	2	1	10
	*D*	2	5	
	*P*	0	0	
2.4. Spontaneous abortion	Unlikely	1	0	1
	Definitely unlikely	0	0	
	possible	0	0	
2.5. Neonatal death	*U*	0	0	2
	*D*	0	1	
	*P*	0	1	
2.6. Pre-eclampsia	Unlikely	1	0	1
	Definitely unlikely	0	0	
	possible	0	0	
2.7. Septicaemia with empetigo	*U*	0	0	1
	*D*	0	1	
	*P*	0	0	
2.8. Tendinitis	Unlikely	1	0	1
	Definitely unlikely	0	0	
	possible	0	0	
***Subtotal SAEs***		***18***	***15***	***33***
**TOTAL (SAEs and AEs)**	**36**		**25**	**61**

*U = Unlikely related, D = Definitely unrelated, P = Possibly related; IUFD = Intrauterine Foetal death;*

§ *Mainly mild/moderate severity; PROM = Premature rupture of membranes.*

Thirty three AEs were defined as SAEs (Table 4) and included 9 preterm deliveries and 10 caesarean sections. Two preterm deliveries in the CTX arm were classified as possibly related to the study drug by the attending physicians who performed the causality assessment, while 4 were unlikely to be related. Both patients were HIV negative and the reason for considering them as SAEs was related to the hospitalization.

### Efficacy Outcomes

There were only 2 patients on the CTX arm and 1 on the SP arm who had malaria positive peripheral smears at the end of follow up of the 347 pregnant women recruited. During the screening phase no patients were declared ineligible due to confirmed clinical malaria (with positive peripheral smear). Among the women screened, there were only 4 out of the 421 that were found to have malaria infection.

Among the placenta blood samples collected on filter paper and analysed by PCR, only 1 sample out of 214 was positive for malaria. Placenta histopathology was also performed and only one sample out of 214 had malaria pigment. This positive sample by histopathology was the same as the placenta blood positive by PCR analysis.

## Discussion

For all HIV-infected adults, including pregnant women, with CD4 cell counts below a given threshold, WHO recommends the use of CTX to prevent opportunistic infections [37]. However, the recommendation also states that pregnant women should not receive SP as IPTp together with CTX prophylaxis. There is evidence that CTX is also an effective antimalarial [33] [38], though the effect of daily CTX prophylaxis during pregnancy on malaria and birth outcomes has not been investigated in HIV-infected or uninfected pregnant women [33] [39]. This was the reason for carrying out in Choma, Zambia, the clinical trial described here.

The trial was aimed at determining the protective efficacy of daily CTX against malaria, both in HIV infected and uninfected pregnant women. Nevertheless, after starting the trial, it became obvious that the risk of malaria infection in the study area had decreased substantially, to the extent that that the trial could not answer the primary question on the protective efficacy of CTX prophylaxis. Therefore, the trial was prematurely terminated though a substantial number of pregnant women had already been recruited. This provided the opportunity of investigating the safety of CTX prophylaxis in an area of extremely low malaria transmission.

Short term treatments with CTX (10–14 days) have been associated with birth defects when administered during the first trimester [40]. In this study, CTX was administered in the second and third trimester and pregnant women were prospectively followed up. Antifolates such as trimethoprim have been known to cause folate deficiency during pregnancy [41] and dietary folate deficiency during the last two trimesters of pregnancy has been linked to preterm delivery and maternal anaemia [42]–[44]. However, in our study daily CTX was not associated with a higher occurrence of adverse pregnancy outcomes and was well tolerated, irrespective of the HIV status. Besides pregnancy related AEs, the other most commonly reported AEs and SAEs were infections and infestations. There was no observable difference in relation to the severity assessment between the study arms and the causality link patterns of the AEs on the CTX arm were comparable to those on the SP arm. Though no birth defect was reported, the sample size was small and probably inadequate to capture rare events, particularly those not immediately visible such as congenital heart disease and other internal organ related defects.

The rate of preterm deliveries using the LMP did not differ between the CTX (combined group of HIV negative and positive mothers) and SP arm. Similarly, the occurrence of preterm deliveries, stillbirths, neonatal mortality, spontaneous abortions and low birth weight among the HIV uninfected and infected with CD4 count ≥200 cells/µl women did not differ between study arms. For both study arms and irrespective of HIV status, the majority of the infants had birth weights consistent with full term delivery. These results are comparable to the findings from a cohort study conducted in the same study area [45] which observed still birth and neonatal death rates of about 2%, and a higher preterm delivery rate (17%) for SP than that (2.1%) observed in this study. However, our findings are based on the sub-analysis of a sample size not sufficiently powered to interpret between group comparisons but rather intended to be purely descriptive of the outcomes. Nevertheless, even if the planned number of pregnant women had been recruited, the study may not have been able to detect rare adverse events. It is important to note that as our findings here do not show an increase in safety risks associated with CTX, we do not suggest that they are conclusive of evidence of absence as explained by Altman et al [46].

After stopping this trial, a similar trial (COTRIMAL – ClinTrialsGov number: NCT 01053325) was set up in an area of high malaria transmission and data are currently being analysed. Therefore, more information on the safety and efficacy of CTX against malaria will be made available in due course.

### Conclusions

Exposure to CTX during the latter part of pregnancy may not be associated with increased safety risks when compared to SP. However, considering the increasing resistance to SP, which is the drug currently used for IPTp, and the need to prevent opportunistic infections in HIV infected pregnant women by administering daily CTX, there is need of exploring the possible role CTX may have in preventing malaria in pregnant women, both HIV infected and uninfected.

## Supporting Information

Checklist S1 **CONSORT Checklist.**
(DOC)

Protocol S1 **Trial Protocol.**
(DOC)
