# Supplementary material for: Safety of Daily Co-Trimoxazole in Pregnancy in an Area of Changing Malaria Epidemiology: A Phase 3b Randomized Controlled Clinical Trial
Source: PLoS One. 2014 May 15;9(5):e96017. doi: 10.1371/journal.pone.0096017 (PMC4022666; doi:10.1371/journal.pone.0096017)
Supplement: Protocol S1 — Trial Protocol. (DOC) [file pone.0096017.s002.doc]

**
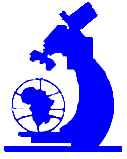

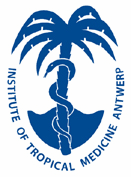
**

# Study Protocol

# A clinical Trial to establish

# The Effectiveness of Daily Co-trimoxazole Prophylaxis

# For Prevention of Malaria

# in Pregnancy

**Protocol version 1.1:**  01 October 2008

**Protocol ID:** MalCotrim in Pregnancy (MALC 008)

**Principal Investigator:** Dr. Christine Manyando

**Sponsor**: Institute of Tropical Medicine, Antwerp, Belgium

**Donor:** The Belgian Development Cooperation in the framework of an Institutional collaboration between the TDRC in Ndola and ITM in Antwerp

**Study drug:** Cotrimoxazole

# Table of Contents

1 Table of Contents [2](#__RefHeading___Toc210709472)

2 Approvals [4](#__RefHeading___Toc210709473)

3 List of Abbreviations [5](#__RefHeading___Toc210709474)

4 Project Synopsis [6](#__RefHeading___Toc210709475)

5 Background [8](#__RefHeading___Toc210709476)

Top Ten causes of Mortality (All ages) 2006-2007 [9](#__RefHeading___Toc210709477)

*Total deaths per 1000 admissions [10](#__RefHeading___Toc210709478)

Top Ten Causes of Morbidity (Under fives) [10](#__RefHeading___Toc210709479)

Top Ten causes of Mortality (Under fives) [11](#__RefHeading___Toc210709480)

Total deaths per 1000 admissions [11](#__RefHeading___Toc210709481)

6 Rationale [13](#__RefHeading___Toc210709482)

7 Study Aim [14](#__RefHeading___Toc210709483)

8 Objectives [14](#__RefHeading___Toc210709484)

8.1. Primary objective [14](#__RefHeading___Toc210709485)

8.2. Secondary objectives [14](#__RefHeading___Toc210709486)

8.3. Exploratory objectives [14](#__RefHeading___Toc210709487)

9 Study Design [15](#__RefHeading___Toc210709488)

10 Sample size [16](#__RefHeading___Toc210709489)

11 Study site [18](#__RefHeading___Toc210709490)

11.1. Overview [18](#__RefHeading___Toc210709491)

11.2. Socio-Economic Profile [19](#__RefHeading___Toc210709492)

12 Training of study personnel [19](#__RefHeading___Toc210709493)

13 Study procedures [20](#__RefHeading___Toc210709494)

13.1. Screening [20](#__RefHeading___Toc210709495)

13. 1. 1. Inclusion criteria [20](#__RefHeading___Toc210709496)

13. 1. 2. Exclusion criteria [20](#__RefHeading___Toc210709497)

13. 1. 3. Baseline characteristics [21](#__RefHeading___Toc210709498)

13.2. Laboratory procedures [21](#__RefHeading___Toc210709499)

13. 2. 1. HIV testing [21](#__RefHeading___Toc210709500)

13. 2. 2. Blood smears for malaria [22](#__RefHeading___Toc210709501)

13. 2. 3. Dried blood spots for P. falciparum genotyping [22](#__RefHeading___Toc210709502)

13.5.4 RPR [23](#__RefHeading___Toc210709503)

13.3. Enrolment [23](#__RefHeading___Toc210709504)

13. 3. 1. Randomisation [23](#__RefHeading___Toc210709505)

13. 3. 2. Treating the patient [24](#__RefHeading___Toc210709506)

13.4. Adverse Events [24](#__RefHeading___Toc210709507)

13. 4. 1. Definition of an adverse event [24](#__RefHeading___Toc210709508)

13. 4. 2. Severity, relationship of event to study drug, and outcome [24](#__RefHeading___Toc210709509)

13. 4. 3. Definition of a serious adverse event [26](#__RefHeading___Toc210709510)

13. 4. 4. Follow-up ANC procedures [26](#__RefHeading___Toc210709511)

13. 4. 5. Breakthrough malaria episodes [27](#__RefHeading___Toc210709512)

13. 4. 6. Concomitant treatment [27](#__RefHeading___Toc210709513)

13. 4. 7. Patient withdrawal/ discontinuation [27](#__RefHeading___Toc210709514)

13.5. Delivery procedures [27](#__RefHeading___Toc210709515)

13. 5. 1. PMTCT [28](#__RefHeading___Toc210709516)

13. 5. 2. Placental smears and histology [29](#__RefHeading___Toc210709517)

Definitions of placental malaria [29](#__RefHeading___Toc210709518)

13.6. Mother and infant follow-up procedures [29](#__RefHeading___Toc210709519)

14 Safety monitoring [30](#__RefHeading___Toc210709520)

14.1. Data Safety Monitoring Board (DSMB) [30](#__RefHeading___Toc210709521)

15 Case Report Form (CRF) [30](#__RefHeading___Toc210709522)

15.1. Presentation of the CRF [30](#__RefHeading___Toc210709523)

15.2. Study protocol and related documentation [30](#__RefHeading___Toc210709524)

15.3. Record retention [31](#__RefHeading___Toc210709525)

16 Monitoring and quality assurance [31](#__RefHeading___Toc210709526)

17 Data Management [31](#__RefHeading___Toc210709527)

17.1. Data Handling [31](#__RefHeading___Toc210709528)

17.2. Data analysis plan [32](#__RefHeading___Toc210709529)

17.3. Outcome Definitions [33](#__RefHeading___Toc210709530)

17. 3. 1. Primary outcomes [33](#__RefHeading___Toc210709531)

17. 3. 2. Secondary outcomes [33](#__RefHeading___Toc210709532)

18 Limitations [33](#__RefHeading___Toc210709533)

19 Timeline [34](#__RefHeading___Toc210709534)

20 Ethical considerations [34](#__RefHeading___Toc210709535)

20.1. Study design [34](#__RefHeading___Toc210709536)

20.2. Informed consent procedures [35](#__RefHeading___Toc210709537)

20.3. Confidentiality [35](#__RefHeading___Toc210709538)

20.4. Ethical review [35](#__RefHeading___Toc210709539)

20.5. Potential risks [35](#__RefHeading___Toc210709540)

20.6. Anticipated benefits [36](#__RefHeading___Toc210709541)

20.7. Confidentiality issues [36](#__RefHeading___Toc210709542)

21 Appendices [37](#__RefHeading___Toc210709543)

21.1. Appendix 1: Dubowitz Test for Gestational Age Assessment [38](#__RefHeading___Toc210709544)

21.2. Appendix 2: Major and Minor Birth Defects Recording [40](#__RefHeading___Toc210709545)

# Approvals

**Protocol approved by:**

**_____________________________Principal Investigator ____/_____/_____**

Christine Manyando, MD, Dip. Epid & Biostat., Cert. Malariology

Tropical Diseases Research Centre,

P. O. Box 71769,

NDOLA, ZAMBIA

Tel: 260 212 621732

Fax: 260 212 621732 or 260 212 620737

E-mail: [cmanyando@yahoo.com](mailto:cmanyando@yahoo.com) or [manyando@zamnet.zm](mailto:manyando@zamnet.zm)

**_________________________________Co-Investigator ____/_____/_____**

Eric Njunju, Bsc Msc

Tropical Diseases Research Centre,

P. O. Box 71769,

NDOLA, ZAMBIA

Tel: 260 212 621112

Fax: 260 212 621732 or 260 212 620737

e-mail: [enjunju@yahoo.com](mailto:enjunju@yahoo.com)

**_**

**________________________________Co-Investigator ____/_____/_____**

Rhoda Mkandawire, MD MPH

Choma District Health Office,

P. O. Box 630741,

CHOMA, ZAMBIA

Tel: 260 213 320370 Or 260 213 320324

Fax: 260 213 320352

e-mail: [fumukazi@yahoo.co.uk](mailto:fumukazi@yahoo.co.uk)

**__________________________________Study Coordinator____/_____/_____**

Jean-Pierre Van geertruyden MD PhD

Dienst Parasitologie, Unit epidemiologie

Instituut voor tropische geneeskunde

Nationalestraat 155

B2000 Antwerpen

Tf ++32 32476363

Tf ++32 484618493

e-mail: [jpvangeertruyden@itg.be](mailto:jpvangeertruyden@itg.be)

**_____________________________________Study Director ____/_____/_____**

Prof. Umberto D’Alessandro, MD PhD

Dienst Parasitologie, Unit epidemiologie

Instituut voor tropische geneeskunde

Nationalestraat 155

B2000 Antwerpen

Tf ++32 32476354

e-mail: [UDAlessandro@itg.be](mailto:UDAlessandro@itg.be)

# List of Abbreviations

ACT Artemisinin-based Combination Therapy

AIDS Acquired immunodeficiency syndrome

ANC Antenatal Care

AZT azitrimycine

CRF Case Report Form

CTX Cotrimoxazole

DSMB Data Safety Monitoring Board

EDD Expected date of delivery

EIA Enzyme immunoassay

FDA Food and Drug Administration

GCP Good Clinical Practice

Hb Haemoglobin

HIV Human Immunodeficiency Virus

ICH International Conference on Harmonisation

IEC Independent Ethics Committee

IPT Intermittent Preventive Treatment

IRB Institutional Review Board

ITN InsecticideTreated bedNet

LBW Low Birth Weight

LMP Last menstrual Period

MCH Mother and Child Health Care

NVP Nevirapine

PMTCT Prevention of Mother To Child Transmission

PRF Patient Record Form

RBM Roll Back Malaria

RPR Rapid Plasma Reagin

SAE Serious Adverse Event

SP Sulfadoxine Pyrimethamine

TDRC Tropical Diseases Research Centre

VCT Voluntary Counselling and Testing

VDRL Venereal Disease Reference Laboratory

WHO World Health Organization

# Project Synopsis

**Title of study:**

A Clinical Trial to establish the Effectiveness of Daily Cotrimoxazole Prophylaxis for Prevention of Malaria In Pregnancy

**Study aim:**

To establish the safety and efficacy of daily CTX in preventing malaria infection during pregnancy and its consequences on the offspring both in HIV-infected and uninfected pregnant women.

**Objectives:**

The **primary objective** is to establish that co-trimoxazole prophylaxis is not inferior to SP prophylaxis in reducing placental parasitaemia in both HIV negative and HIV positive pregnant women

- The **secondary objectives** are:
- To evaluate efficacy of CTX prophylaxis in reducing malaria peripheral parasitaemia.
- To evaluate efficacy of CTX prophylaxis in reducing perinatal mortality and in improving birth weight
- To establish the safety of CTX prophylaxis on the offspring by measuring the gestational age at delivery and birth weight.
- To compare the efficacy profile of CTX prophylaxis to that of SP intermittent preventive treatment.
- To compare the safety profile of CTX prophylaxis to that of SP intermittent preventive treatment.

These shall be undertaken in both HIV negative and HIV positive pregnant women independently.

**Exploratory objectives**

Information will be collected concurrently in order to monitor for potential elevated risk after prophylaxis with co-trimoxazole and SP during pregnancy irrespective of HIV serostatus. This information will comprise of spontaneous abortions (</=28 weeks gestation), preterm deliveries (<37 completed weeks), neonatal mortality (within 28 days after birth), maternal mortality (Up to 6 weeks following delivery) and major and minor birth defects (at birth and up to 6 weeks).

**Study population:**

The study population will comprise of pregnant women asymptomatic of malaria at recruitment, who will be recruited in the ratio of 1:1 for CTX prophylaxis and SP prophylaxis. A total of 2280 pregnant women, 800 in each arm of the HIV negative, and 680 in the arm of the HIV positive with CD4 count >200 cells/mm^3^ and 100 in total of the HIV positive with CD4 count <200 cells/mm^3^ will be recruited. These will be recruited from the Choma district in Zambia.

**Inclusion criteria**

- Confirmed pregnancy (through palpable fundus and/ or positive pregnancy test)
- Gestational age between 16 and 28 weeks.
- Signed or right thumbprint marked informed consent by patient (or parent/ guardian if patient is less than 18 years of age)
- Should have no symptoms consistent with malaria
- Willingness to deliver at the health facility
- Willingness to adhere to all requirements of the study (including HIV-1 testing)

**Exclusion criteria**

- History of allergy to study drugs, or previous history of allergy to sulpha drugs
- History or presence of major illnesses likely to influence pregnancy outcome including diabetes mellitus, severe renal or heart disease, or active tuberculosis, prior to randomization;
- Any significant illness that requires hospitalization;
- Intent to move out of the study catchment’s area before delivery or deliver at relative’s home out of the catchment’s area;
- Prior enrolment in the study or concurrent enrolment in another study
- Severe anaemia (Hb<7 g/dl)
- Previous history of unfavourable pregnancy outcome: pre-eclampsia, caesarean section, stillbirth.

# Background

**Malaria in Pregnancy**

Malaria remains a major contributor of the disease burden in Sub-Saharan Africa. Approximately 90% of global cases of malaria occur in Sub-Saharan Africa, with pregnant women and children under 5 years of age, representing the most vulnerable population ([[1]](#endnote-2)). Annually, around 25 million of pregnant women are at risk of *Plasmodium falciparum* infection during their pregnancy. *Plasmodium falciparum* infection in pregnancy leads to parasite sequestration in the maternal placental vascular space, with the consequence of increased risks of abortion, stillbirth, prematurity, intrauterine growth retardation and maternal anaemia. It is associated with increased risk of low birth weight (LBW) and perinatal, neonatal and infant mortality.

The symptoms of malaria during pregnancy and complications differ with the intensity of malaria transmission and thus with the level of immunity the pregnant woman has acquired during subsequent pregnancies. In low transmission areas all pregnant women have little or no pre-existing immunity and malaria can evolve towards severe disease with a high risk of maternal and perinatal mortality. Foetal and perinatal loss can be as high as 60-70% ([[2]](#endnote-3), [[3]](#endnote-4), [[4]](#endnote-5), [[5]](#endnote-6)) . In highly endemic areas, where multigravidae have acquired malaria immunity, malaria infection is still associated with maternal anaemia, LBW and stillbirth ([[6]](#endnote-7),[[7]](#endnote-8)).

The mean birth weight of infants born by mothers with placental malaria is reduced by 55-310 g ([[8]](#endnote-9),[[9]](#endnote-10),[[10]](#endnote-11),[[11]](#endnote-12))***.*** Placental malaria is responsible for up to 35% of preventable LBW in malaria-endemic area ([[12]](#endnote-13)). Moreover, LBW is more frequent in primigravidae with placental malaria as compared to those without ([[13]](#endnote-14)) and it is the most important cause of infant mortality, responsible in Africa for at least 13% of all infant deaths ([[14]](#endnote-15),[[15]](#endnote-16)). A recent review analyzing the malaria population attributable risk for anaemia [3-15%], LBW [8-14%] and infant mortality [3-8%] estimated that each year between 75,000 and 200,000 infant deaths are associated with malaria infection in pregnancy ([[16]](#endnote-17),[[17]](#endnote-18)).

WHO recommends a package of interventions for the prevention and control of malaria during pregnancy. This comprises Intermittent Preventive Treatment (IPT) to address the heavy burden of asymptomatic infections among pregnant women residing in areas of moderate or high transmission of *P. falciparum*, use of insecticide treated nets (ITNs) ([[18]](#endnote-19)), and access to effective case management for malaria illness and anaemia. There is evidence that prophylaxis with efficacious antimalarials can reduce the incidence of placental parasitaemia, LBW, and maternal anemia ([[19]](#endnote-20)).

**Malaria and HIV in pregnancy**

LBW is known to be the most important risk factor for infant mortality ([[20]](#endnote-21)). Anaemia, malnutrition and HIV infection are also common events in malaria endemic areas and contribute to LBW. HIV infection in pregnancy confers additional risk for higher frequency and higher density of malaria during pregnancy ([[21]](#endnote-22)). Furthermore,

observations on pregnant mothers demonstrate an increased risk for malaria infection related to HIV infection ([[22]](#endnote-23)) The risk increment is more pronounced in multigravidae than in primigravidae, indicating that HIV-1 hinders the maintenance of immunity. ([[23]](#endnote-24), [[24]](#endnote-25), [[25]](#endnote-26)). A 2-fold increase in malaria incidence was also observed. Available data has been recently reviewed and summarized ([[26]](#endnote-27)). The relative risk for malaria infection in HIV-1 infected women has been estimated during pregnancy (risk ratio= 1.58), at delivery (RR = 1.65), and for placenta malaria (RR =1.66). HIV-1 infection was also associated with lower birth weight ([[27]](#endnote-28)), higher infant mortality, a 4-fold greater risk of malaria attack in the new-born ([[28]](#endnote-29)).

The increased risk of placental malaria in HIV infected mothers was also associated with a higher post-natal mortality ([[29]](#endnote-30)). The risk of anaemia is beyond the degree that would be expected from infections with malaria or HIV alone, suggesting a synergistic interaction between HIV and malaria, placing dually infected women at very high risk of developing severe anaemia (42, 44).

In HIV infected pregnant women, it has been shown that HIV-1 infection decreases the efficacy of SP intermittent preventive treatment during pregnancy although 2 or more doses of SP given during pregnancy (in the 2^nd^ and 3^rd^ trimesters) still reduce peripheral parasitaemia, placental infections and maternal anaemia including severe anaemia, and increase birth weights ([[30]](#endnote-31), [[31]](#endnote-32))*.*

**Burden of malaria in Zambia**

In Zambia, malaria continues to be a major public health problem, exacting its largest toll on children less than 5 years of age and pregnant women. A trend analysis conducted between 1999 and 2003 showed a proportional increase in the number of malaria cases and deaths contributing to 37% of all the diagnoses and 45% to the top ten diagnoses ([[32]](#endnote-33)) as evidenced by the Health Management Information Systems ([[33]](#endnote-34)). The malaria incidence rate increased from 121.5 cases per 1000 population in 1976,; to 482.0 per 1000 population in 2003. These high incidence rates were predominantly evident among the pregnant women and children under five, contributing to the already high infant and maternal mortality rates by 40% and 20% respectively (The infant mortality rate was 104/1000 live births in 2004 and the maternal mortality rate was 750 per 100 000 live births in 2000) ([[34]](#endnote-35)).

### Top Ten causes of Mortality (All ages) 2006-2007

|  | **2007 Qtr 1 & 2** | | | **2006** | | |  |
| --- | --- | --- | --- | --- | --- | --- | --- |
|  |  | **# Dead*** | **%** | **Disease** | **# Dead*** | **%** | |
| **1** | **Malaria** | **102** | **16.9** | **AIDS** | **192** | **16.7** |  |
| 2 | Pneumonia | 78 | 12.9 | **Malaria** | **185** | **16.1** |  |
| **3** | **AIDS** | **75** | **12.4** | Diarrhoea Non-Bloody | 109 | 9.5 |  |
| 4 | Cardio – vascular | 42 | 6.9 | Pneumonia | 97 | 8.4 |  |
| 5 | Perinatal | 33 | 5.5 | Any perinatal conditions | 83 | 7.2 |  |
| 6 | Anaemia | 32 | 5.3 | Tuberculosis | 75 | 6.5 |  |
| 7 | Tuberculosis | 30 | 5.0 | Malnutrition(PEM) | 69 | 6.0 |  |
| 8 | Diarrhoea (Non-Bloody) | 29 | 4.8 | Cardio – vascular disease | 51 | 4.4 |  |
| 9 | Malnutrition (PEM) | 28 | 4.6 | Anaemia | 50 | 4.3 |  |
| 10 | Meningitis | 17 | 2.8 | Respiratory infection | 21 | 1.8 |  |

### ***Total deaths per 1000 admissions**

- Malaria is the most common cause of mortality followed by pneumonia in the 1^st^ and 2^nd^ quarter of 2007, while in 2006 AIDS is number one followed by malaria.
- There have been some deaths from non-bloody diarrhoea as number three in 2006 while it was number eight in quarter 1 and 2 in 2007. The underlying cause of these deaths could have been HIV/AIDS related.
- Case fatality rate for pneumonia has decreased from 120 in 2005 to 78 in quarter 1 and 2 of 2007.
- CFR for anaemia remained at 41 deaths for 2004 and 2005 and increased to 50 deaths in 2006 while for quarter 1 and 2 of 2007 at 32 deaths.

### Top Ten Causes of Morbidity (Under fives)

|  | **2007 Quarter 1 and 2** | | | **2006** | | |
| --- | --- | --- | --- | --- | --- | --- |
|  | **Disease** | **Incidence** | **Incidence rate** | **Disease** | **Incidence** | **Incidence rate** |
| **1** | **Malaria** | **24,636** | **509.4** | **Malaria** | **53,847** | **1,140.4** |
| 2 | Respiration infection | 16,696 | 345.2 | Respiration infection | 30,559 | 647.2 |
| 3 | Diarrhoea (non blood) | 4,046 | 83.7 | Diarrhoea (non bloody) | 10,235 | 216.8 |
| 4 | Eye infections | 2,848 | 58.9 | Eye infections | 9,836 | 208.3 |
| 5 | Skin infections | 2,673 | 55.3 | Skin infections | 5,108 | 108.2 |
| 6 | Respiratory infection : pneumonia | 1,663 | 34.4 | Ear/ nose/ throat infections | 2,804 | 59.4 |
| 7 | Ear/ nose/ throat infections | 1,560 | 32.3 | Respiratory infection : pneumonia | 2,680 | 56.8 |
| 8 | Skin Disease(not infectious) | 1,011 | 20.9 | Skin Diseases (not infectious) | 2,163 | 45.8 |
| 9 | Intestinal worms | 547 | 11.3 | Diarrhoea :bloody (suspected dysentery) | 1,548 | 32.8 |
| 10 | Diarrhoea :bloody (suspected dysentery) | 526 | 10.9 | Intestinal worms | 1,308 | 27.7 |

- Malaria has been the major cause of morbidity through out the years under review and has been increasing from 46,838 cases in 2004 to 53,847 cases in 2006 and 24,636 in 2007 quarter 1 and 2.
- Respiratory infection non-pneumonia, diarrheoa non-blood, eye infections and skin infections have maintained there positions as number two, three, four and five respectively over the years under review. All these diseases have shown an increase in the years under review.
- Pneumonia increased from 2,575 cases in 2004 to 3,076 in 2005 and decreased to 2,680 in 2006 and at 1,560 cases in quarter 1 and 2 in 2007.

### Top Ten causes of Mortality (Under fives)

|  | **2007 quarter 1 and 2** | | | **2006** | | |
| --- | --- | --- | --- | --- | --- | --- |
|  | **Disease** | **No Dead*** | **%** | **Disease** | **# Dead*** | **%** |
| **1** | **Malaria** | **63** | **23.5** | **Malaria** | **86** | **19.3** |
| 2 | . Pneumonia | 45 | 16.8 | Any perinatal conditions | 83 | 18.7 |
| 3 | Any perinatal conditions | 33 | 12.3 | . Malnutrition | 68 | 15.3 |
| 4 | Malnutrition | 26 | 9.7 | Diarrhoea Non-Bloody | 55 | 12.4 |
| 5 | Diarrhoea Non-Bloody | 15 | 5.6 | Pneumonia | 52 | 11.7 |
| **6** | **AIDS** | **14** | **5.2** | Anaemia | 26 | 5.8 |
| 7 | Anaemia | 13 | 4.9 | **AIDS** | **23** | **5.2** |
| 8 | Respiratory infection: non-pneumonia | 4 | 1.5 | Respiratory infection: non-pneumonia | 8 | 1.8 |
| 9 | Meningitis | 4 | 1.5 | Tuberculosis | 3 | 0.7 |
| 10 | Diarrhoea: blood(suspected | 1 | 0.4 | Poisoning | 3 | 0.7 |

### **Total deaths per 1000 admissions**

- Malaria stands out as the number one cause of mortality in the years under review except for 2005 in which pneumonia stands as number one.
- Perinatal conditions remained as number two cause of mortality from 2004 to 2006 in under five. While for quarter 1 and 2 in 2007 is number two.
- Anaemia has shown an increase trend in the years under review although it is not among the top five causes of mortality in under five.
- Diarrhoea non-bloody has shown an increase trend from 32 deaths in 2004 to 55 deaths in 2006 and 15 deaths in quarter 1 and 2 of 2007.
- Protein Energy Malnutrition (PEM) has remained on the top 5 causes of Mortality for the past three years and quarter 1 and 2 of 2007.

**Malaria prevention in Pregnancy in Zambia**

Malaria prevention in pregnancy relies on use of ITNs and use of IPT during pregnancy. The Zambia National Malaria Indicator Survey conducted in 2006, revealed that 24% of all women aged 15-49 years slept under a mosquito net the night before the survey and 21% slept under an ITN. For pregnant women, the percentage sleeping under a mosquito net (33%) and ITNs (23%) were higher than the percentages for all women (pregnant and non-pregnant). Seventy-seven percent of mothers reported taking an anti-malarial drug for prevention during their last pregnancy. Seventy-five percent (75%) of mothers received the antimalarial drug during routine antenatal clinic (ANC) visit. Of mothers who took an antimalarial drug, 62% took recommended two or more doses of IPT. Similarly, 61% of mothers received two doses of IPT, at least one of which was during an ANC visit (73).

**The potential role of Cotrimoxazole for improved birth outcomes**

Several studies in Zambia and Uganda demonstrated that daily CTX prophylaxis, a sulfa drug as SP, is effective in reducing mortality and morbidity in HIV infected individuals ([[35]](#endnote-36),[[36]](#endnote-37)). This was observed despite high in-vitro resistance of common bacterial infections to co-trimoxazole (60-80%) and irrespective of age and CD4 count.

Evidence is available to demonstrate that antibiotic therapy during pregnancy might be beneficial and impacts positively to the reduction to some extent of adverse pregnancy outcomes such as UTI, toxoplasmosis, malaria, systemic infections like pneumonia, lower genital tract infections like Chlamydia and gonorrhoea. ([[37]](#endnote-38)). CTX prophylaxis significantly improves birth outcomes in HIV-infected women with a CD4 count <200 cells per mm^3^, with reduction of chorionamnionitis, prematurity and neonatal mortality..A recent study, in Zambia conducted using historical controls concluded that antenatal provision of cotrimoxazole was beneficial for HIV-infected pregnant women with low CD4 cell counts but not in women with ≥200 CD4/µL ([[38]](#endnote-39)). However, this study was carried out in an area with an extremely low risk of malaria infection; CTX may have had a different impact if malaria transmission was either holo or hyperendemic. As far as safety is concerned, a recent review of the use of CTX during pregnancy was unable to substantiate the increased risk of neonatal jaundice or kernicterus with the use of CTX while data on the risk of birth defects were inconclusive ([[39]](#endnote-40)). As the advantages of CTX seem to be clear in all HIV infected with CD4 <200/µL, including pregnant women, WHO now recommends daily co-trimoxazole, in addition to ARV, to prevent opportunistic infections, regardless of the background prevalence of CTX microbial resistance ([[40]](#endnote-41)).

Concurrent administration of SP and CTX has been associated with a substantially increased incidence of severe adverse reactions in HIV-infected patient. Therefore, WHO has promoted CTX prophylaxis (2 tablets of single strength 400mg of sulphamethoxazole and 80 mg trimethoprim per day) as an alternative to sulfadoxine-pyrimethamine for the intermittent preventive treatment in immuno-compromised (CD4 count < 200) HIV-infected pregnant women (Error: Reference source not found).

Unfortunately, there is no information on the effectiveness of daily CTX for preventing neither malaria infection (placental parasitaemia) nor its consequences (maternal anaemia and low birth weight) in pregnancy. In the past, CTX, has been used to treat malaria in children and daily use of CTX by non-pregnant HIV-infected adults has been associated with a 70% reductions of the incidence of clinical malaria ([[41]](#endnote-42),[[42]](#endnote-43),[[43]](#endnote-44))

.The focus of this study is the malaria related outcome in pregnancy and we will target both HIV infected and non-infected pregnant women with CD4≥ 200/µL, assuming that CTX is not inferior to SP in reducing placental parasitaemia

Such information is urgently needed in order to assist to issue guidelines on intermittent preventive treatment in pregnant women (Figure 1)([[44]](#endnote-45)).

**Figure 1: Suggested priorities for future research and surveillance (*Lancet Infect Dis* 2006;6:100-111**)

# Rationale

Presently, sulfadoxine-pyrimethamine (SP) is the only antimalarial treatment for which data on efficacy and safety for IPT is available. The WHO recommends that at least 2 doses of SP, at least one month apart, are given after the first trimester during regularly scheduled antenatal visits. Currently, there is a documented increase in SP resistance and the other antimalarials are still being studied for safety and efficacy even for the treatment of uncomplicated malaria in pregnancy. CTX (Co-trimoxazole) could be an alternative for SP in reducing malaria and malaria related morbidity and mortality in pregnancy.

# Study Aim

To establish the safety and efficacy of daily CTX in preventing malaria infection during pregnancy and its consequences on the offspring both in HIV-infected and uninfected pregnant women.

# Objectives

## Primary objective

To establish that co-trimoxazole prophylaxis is not inferior to SP prophylaxis in reducing placental parasitaemia in both HIV negative and HIV positive pregnant women

## Secondary objectives

- To evaluate efficacy of CTX prophylaxis in reducing malaria peripheral parasitaemia.
- To evaluate efficacy of CTX prophylaxis in reducing perinatal mortality and in improving birth weight
- To establish the safety of CTX prophylaxis on the offspring by measuring the gestational age at delivery and birth weight.
- To compare the efficacy profile of CTX prophylaxis to that of SP intermittent preventive treatment.
- To compare the safety profile of CTX prophylaxis to that of SP intermittent preventive treatment.

These shall be undertaken in both HIV negative and HIV positive pregnant women.

## Exploratory objectives

Information will be collected concurrently in order to monitor for potential elevated risk after prophylaxis with co-trimoxazole and SP during pregnancy irrespective of HIV serostatus. This information will comprise of spontaneous abortions (</=28 weeks gestation), preterm deliveries (<37 completed weeks), neonatal mortality (within 28 days after birth), maternal mortality (Up to 6 weeks following delivery) and major and minor birth defects (at birth and up to 6 weeks).

# Study Design

The study is designed as a two parallel open-label Randomised Clinical Trial (RCT) comparing CTX and SP in HIV negative and HIV positive with CD4 count above or equal to 200/µl. This parallel design was chosen to permit sufficient power in each group as HIV might interact with CTX efficacy.

Women on CTX and/ or ARV treatment or having a CD4 count under 200µl will be included in a prospective observational cohort.

In Zambia, a programme offering HIV testing facilities at the health facilities already exists. CTX prophylaxis was introduced in HIV positive women CD4 count under 200/µl as part of routine Antenatal Care in November, 2003 .

All women who will accept to be tested for HIV at the recruiting health facilities and are willing to participate in the study shall give a signed informed consent. All women who will test positive shall have their CD4 counts measured. At enrolment all women who will have CD4 <200 cells/mm^3^ and are 16 weeks gestation and above will receive 2 tablets of CTX/daily. Both HIV-infected women with a CD4 count>200 cells/ mm^3^ and those HIV-negative will be randomised to either CTX daily or SP IPT. The randomisation will be stratified by HIV status.

HIV-infected women with CD4 count<200 cells/mm^3^ who are already on CTX and/or ARV treatment but are complying with all other inclusion and exclusion criteria will be recruited in an observational study after they give a signed informed consent. Information on clinical malaria, including treatments received and number of CTX taken will be collected. All pregnant women with HIV infection with CD4 cell counts less than 200 /µl, who are in second and third trimesters of gestation shall receive 2 single-strength tablets (400mg of sulphamethoxazole and 80mg of trimethoprim) daily and Antiretroviral treatment as per standard guidelines.

HIV-infected women with CD4 count<200 cells/mm^3^ who present themselves only at delivery at the health facilities will be receive ARVs to prevent mother to child transmission of HIV (PMTCT). If they are willing to participate and sign an informed consent, information on clinical malaria, including treatments received (including SP IPT and CTX) will be collected retrospectively using a structured questionnaire.

# Sample size

Data from both study sites will be pooled (about 5 km distance).

**HIV negative sample size calculation**

Sample size for this study was determined by simulation with the following assumptions and requirements:

(1) CTX prophylaxis is considered equivalent to SP if the two-sided 95% confidence interval for the prevalence of placental malaria for both SP and CTX, expected to be 10%, lies entirely below 15%

(2) Study conclusions are determined by the decision rule as described above,

(3) Placental malaria prevalence is 10% in both treatment groups,

(4) We require 90% power for the primary hypothesis.

With these assumptions, we need approximately 716 patients/treatment arm. Allowing for a 10% loss to follow-up, a total of 788 women will be randomized to each treatment. The loss to follow up in the Coartem® pregnancy registry located at the same sites was 8%. If we want to obtain this sample size for HIV negative, we‘ll need 27 months to recruit the required sample size.

**HIV infected with CD4≥ 200 sample size calculation**

Sample size for this study was determined by simulation with the following assumptions and requirements:

(1) CTX prophylaxis is considered equivalent to SP if the two-sided 95% confidence interval for the prevalence of placental malaria for both SP and CTX, expected to be 10%, lies entirely below 20%.

(2) Study conclusions are determined by the decision rule as described above,

(3) Placental malaria prevalence is 10% in both treatment groups,

(4) We require 90% power for the primary hypothesis.

With these assumptions, we need approximately 236 patients/treatment arm. Allowing for a 10% loss to follow-up, a total of 260 women will be randomized to each treatment. The loss to follow up in the Coartem® pregnancy registry located at the same sites was 8%. We‘ll need 21 months to recruit the required sample size.

**Overall**

Therefore, we will recruit eligible, HIV negative and HIV infected women with CD4≥200, till the required sample size for HIV negative stratum is reached. In this time span of 27 months we’ll recruit about 1576 HIV negative (=2*788) and 680 HIV infected (=2*340) eligible women.

This overall sample of over 2000 deliveries will permit us to conduct several highly powered sub analyses in the HIV-1 negative and HIV-1 infected group (table). In addition, data from HIV infected and HIV infected with CD4≥200 might be combined if no difference in treatment effect between CTX and SP is found.

**Power for finding secondary outcomes (all calculated with foreseen study sample size)**

|  | **Estimated** | | **Max Difference, within IC 95% limits, accepted between treatment arms** | | **Power with total Sample size needed** | |
| --- | --- | --- | --- | --- | --- | --- |
|  | HIVN | HIVP (≥200CD4/µL) | HIVN | HIVP | HIVN | HIVP |
| Birth weight (g) | 2870 g | 2740 g | 143 g (5%) | 137 g (5%) | 99.9 % | 89.0 % |
| % Hb < 7 g/dl | 5 % | 8 % | 4 % | 6 % | 95.4 % | 82.2 % |
| % Neonatal death within 30 days of birth | 3 % | 5 % | 3 % | 5 % | 93.3 % | 84.8 % |

# Study site

The study will be conducted in Choma District in Southern Province of Zambia. Two sites were chosen: Shampande Clinic and Choma hospital.

Choma was specifically chosen because there is moderate to high transmission of malaria. Both sites were involved in a WHO-monitored Pregnancy Registry of Coartem® for the past four years providing a rich resource of GCP/ GCLP trained staff who are capable of conducting this type of study. Shampande clinic had 690 deliveries in 2007. The Hospital had over 800 deliveries of whom 300 where followed up at the MCH of the hospital. Over 90 percent of the women have their first ANC in the second trimester

| **Parameter** | **Shampande** | **Choma Hospital MCH** | **Total** |
| --- | --- | --- | --- |
| **Catchment population** | 14584 | 4911 | 19495 |
| **New ANC attendees** | 940 | 202 | 1142 |
| **Expected pregnancies** | 788 | 265 | 1053 |
| **Expected deliveries** | 758 | 255 | 1013 |
| **Services provided** | VCT/PMTCT | PMTCT |  |
| **SP available** | X | X |  |
| **Tests for HIV Abbott/Unigold** | X | X |  |
| **CD4 count available** | ---- | X |  |

## Overview


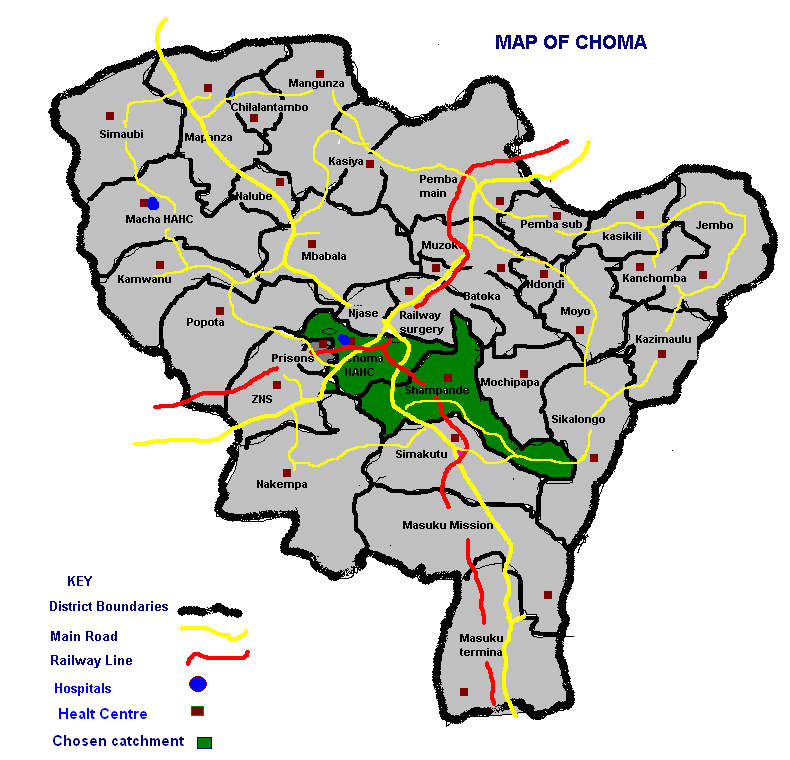


Choma District is situated along the line of rail and the major highway, 289 Km south of Lusaka, Zambia’s capital city and 188 Km north of Livingstone, the provincial capital of Southern Province . It is the centre of Southern province and shares borders with five of the eleven other Districts in the province namely Monze, Namwala, Kalomo, Gwembe and Sinazogwe.

The district covers an area of about 7,300 Km sq. with a population of approximately 266,000 people, which represents a density of 33 per Km sq. Choma District is 1,200 m above the sea level and can be defined as mesoendemic for malaria.

Communication has improved since most Centres now have radio contact including the two proposed recruiting sites. However, today there is omnipresence of mobile telephones.

Choma District has five laboratories at Shampande Clinic, Pemba Main, Mapanza, Choma Hospital and Macha Hospital. These facilities are diagnostic centres for malaria and TB. Radiographic services are available at Choma hospital and Macha mission hospital. The district has twenty eight VCT/PMTCT and three ART centres.

## Socio-Economic Profile

The main occupation is agricultural production i.e. cropping and ranching. Choma is basically a farming area, producing maize and tobacco, and supplies the Country with some prime meat. Most Communities are subsistence farmers, mainly growing maize. The District has few major industries. A good number of industries have folded up, making a negative impact on the social economic status. However, the many Government departments keep the civil service functioning. There are also a lot of self-employed business people in Choma.

Most women and girls have limited education as compared to the male folk. This is as the result of the cultural and traditional background.

Since Choma is centrally situated along the Livingstone – Lusaka road (Great North Road), most long distance drivers have made it their resting place, especially the truck drivers and thus an increase in the temporal relationship with the local women, determining the high risk for HIV infection

Choma has eight high boarding schools namely: Njase girls, Macha girls, Choma, Pemba, Sikalongo, St Marks, Masuku and Jembo respectively. Most pupils come from other towns.

# Training of study personnel

All study staff will undergo extensive training for standardization of procedures before the patient recruitment of patients. This will be accomplished in part by giving didactic sessions to midwives, and laboratory technicians to describe the goal and methods of the study. Familiarization with study equipment, supplies and forms will also occur through these sessions. Further training will occur during the pilot phase of the study when approximately 4 women from each ANC clinic will be enrolled and will undergo all baseline procedures.

# Study procedures

## Screening

All potential study participants shall be recruited from the ANCs in Choma district. A screening log shall be maintained so as to capture in-case the pregnant women who may be unwilling to participate in this study and may constitute a population with systematically different characteristics and outcomes. Hence, in order to assess whether such bias exists, a screening log of key baseline information (age, area of residence, reason for not participating, etc) will be kept. This will help to determine any systematic differences between those who participate and those who choose not to participate.

Women will be recruited in the ANC at booking or subsequent visits from 16 weeks up to 28 weeks. All women presenting to the ANC will be offered the opportunity to undergo VCT as is the current practice at the health facilities in Choma. Women who complete VCT will be screened for the study. At the time of screening, the study will be explained in detail to the woman. The screening project member will determine if the pregnant woman meets all the eligibility criteria. Women who meet all the criteria and are willing to follow all the study procedures will be asked to provide written informed consent in the presence of one member of the study. The informed consent will describe the purpose of the study, the procedures to be followed and the risks and benefits of participation. A copy of the consent form will be given to the women. Each woman screened will be assigned a unique screening number and those who agree to enroll will be assigned a separate unique study number. All HIV-positive women who meet the study eligibility criteria will be offered the opportunity to participate in the study.

### Inclusion criteria

- Confirmed pregnancy (through palpable fundus and/ or positive pregnancy test)
- Gestational age between 16 and 28 weeks.
- Signed or right thumbprint marked informed consent by patient (or parent/ guardian if patient is less than 18 years of age)
- Should have no symptoms consistent with malaria
- Willingness to deliver at the health facility
- Willingness to adhere to all requirements of the study (including HIV-1 testing)

### Exclusion criteria

- History of allergy to study drugs, or previous history of allergy to sulpha drugs
- History or presence of major illnesses likely to influence pregnancy outcome including diabetes mellitus, severe renal or heart disease, or active tuberculosis, prior to randomization;
- Any significant illness that requires hospitalization;
- Intent to move out of the study catchment area before delivery or deliver at relative’s home out of the catchment area;
- Prior enrolment in the study or concurrent enrolment in another study
- Severe anaemia (Hb<7 g/dl)
- Previous history of unfavourable pregnancy outcome: pre-eclampsia, caesarean section, stillbirth.

### Baseline characteristics

If inclusion criteria are met, baseline data including medical history, obstetric history, date of LMP, haemoglobin and behavioural risk factors (such as alcohol intake and smoking ) that may affect the pregnancy outcome will be recorded using Patient Record Forms (PRFs). The socioeconomic status will also be assessed on some key household parameters such as access to clean water supply, toilet facilities, access to electricity and possession of various goods.

Baseline data that will be collected at the time of enrolment will include date of birth, level of education, socioeconomic status based on area of residence, previous child deaths, bed net use, history of recent illnesses including symptoms of AIDS, history of malaria symptoms, and history of recent antimalarial drug use. Within each area of residence, the following household factors will be evaluated: availability of piped water inside dwelling, type of latrine available, and number of other adults and children per dwelling. To facilitate outpatient follow-up, all subjects will be requested to supply the name of the head of household, the home address, directions and a map to help with how to find their home, and the name and addresses of a family, friend, or village community health worker contact.

An initial physical examination will be performed; this will include temperature, pulse, blood pressure, height, weight, fundal height, fetal heart rate, and fetal position. Gestational age will be estimated based upon the woman’s history of last menstrual period and fundal height.

Baseline laboratory work will include thick blood smears of fingerprick sample for quantitation of asexual malaria parasites and hemoglobin (Hb) level. Approximately 50 μL of blood will be placed on filter paper for future *P. falciparum* genotyping .

Once enrolled, the participating pregnant women will be given a visit schedule for follow-up assessments of the mother and baby.

The study assessments will be carried out at the ANC clinics. Home visits will be carried out for any of the participants that do not show up for the scheduled visits. At all scheduled visits, the participants will be provided with transport refunds to facilitate their travel for the visits.

## Laboratory procedures

Details of the laboratory procedures can be found in the Study Procedure Manual.

### HIV testing

Blood will be collected from all women consenting to be tested for HIV. Two rapid HIV tests will be employed on-site within the two clinics: screening will be done with the HIV1+2, using the Abbot Determine™ HIV 1 and 2, an in—vitro, visually read, qualitative immunoassay for detection of antibodies to HIV-1 and HIV-2 in human serum, plasma or whole blood. Confirmation with the Unigold™ test for HIV-1/HIV-2 test (Trinity Biotech PLC, IDA Business Park, Bray, Co Wicklow, Ireland). These tests will be run on the same blood specimen to identify HIV-seropositive women. HIV indeterminate results will be tested again with each test and, if necessary, confirmed by a 3^rd^ rapid test and/or EIA at the coordinating center facilities.

CD4 counts will be determined using CD8 and CD4 fluorochrome labelled antibodies on an aliquot of whole blood using a lyse-no wash method. The absolute numbers of CD4-positive and CD8-positive T lymphocytes will be obtained in a single platform system using true count tubes containing a known number of fluorochrome-integrated polystyrene beads. Samples will be processed by flow cytometry on a CyFlow (Cyflow® Counter, Partec, Germany or Guava EasyCD4 System from Germany) within 3 hours of collection ([[45]](#endnote-46)).

### Blood smears for malaria

Blood will be obtained by fingerstick from pregnant women after informed consent has been obtained and at the time of delivery. If a woman reports to the clinic between scheduled ANC visits with symptoms suggestive of malaria, then a fingerstick will be done to obtain blood for thick smears. After delivery, thick blood smears will be made from the mother by fingerstick, the maternal side of the placenta, and the umbilical cord.

Laboratory diagnosis of malaria will be done by the examination of Giemsa-stained thick blood slide smear. When mixed infection is suspected in the thick smear, a thin film will be made to identify the species of *Plasmodium.* Total parasite count per μL will be quantitated based on a review of high power fields containing 200 white blood cell[[46]](#endnote-47) using a hand tally counter. The parasitaemia in microlitre is calculated by using the formula:

Parasitaemia (/microlitre)= number of parasites x WBC count (8000) *

number leukocytes counted (200)

If >500 parasites have been counted without having reached 200 leukocytes, the count is stopped after completing the reading of the last field and the parasitaemia is calculated according to formula above. If *P.falciparum* gametocytes are seen, a gametocyte count is performed against 1000 leukocytes. A thick smear will be declared negative after viewing high power fields containing 500 white blood cells.

### Dried blood spots for P. falciparum genotyping

Baseline blood will be placed on filter paper, dried, and then stored in a sealed plastic bag with dessicant at –20°C. This will be eventually used for molecular analyses of the highly polymorphic P. falciparum merozoite surface protein-1 by nested PCR assays to be carried out by TDRC with the support of Institute of Tropical Medicine in Belgium.

.

Blood obtained by fingerstick from the mother will be used to measure Hb level at the time of study entry. Hb will be measured once early in the third trimester between weeks 30-34 of pregnancy and immediately after delivery. Hb measurements will be performed using the Hemocue® machine with the use of a control with each assay.

### 13.5.4 RPR

It is part of routine Zambian antenatal assessment to test every pregnant woman for syphilis. The Immutrep Rapid Plasma Reagin (RPR) test from Omega Diagnostics will be used for testing syphilis in plasma or serum. This test is a flocculation test and is modified from the Immutrep VDRL antigen test so as to improve the visual reading of the result.

The test comes as a complete kit and can be performed on heated or unheated samples and can be used in both a semi-quantitative and qualitative manner.

Prior to use, stored frozen samples (-20^o^C) are brought to room temperature. Using a disposable plastic pipette, one drop of plasma or serum is spread evenly over the test circle on the plastic card. One free falling drop of reagent antigen is mixed with the test specimen. The test card is centrifuged at 100 r.p.m for eight minutes and the result visualized directly under a strong light source. A positive result is indicated by the presence of visible flocculation on the card. To maintain test validity, each set of tests is run in parallel with a positive control using a characterized sample of control serum.

The results are read as:

1. Medium to large granules Reactive

2. Finely dispersed aggregates Weak reactive

3. No aggregates, visible grey appearance Non reactive

**(ref: Immutrep RPR. Rapid Plasma Reagin Test Card for the sero-diagnosis of syphilis. Omega Diagnostics Limited)**

The standard treatment for every reactive patient to the RPR test (whether strongly reactive or weakly reactive) is to receive Benzathine Penicillin 2.4 mega units intra muscularly weekly for three weeks. This will be the medication to be provided to study participants. The test is normally repeated a month later to confirm that the test is no longer reactive and patient has been fully treated.

## Enrolment

Enrolment of participants will be done at the two ANC clinics within Choma district already mentioned. The study midwives will identify the potential participants and the research doctors will affirm the processes of informed consent to participate. The study midwives will assist to establish history of malaria morbidity, as well as whether the pregnant women meet the criteria for enrolment.

### Randomisation

HIV pregnant women with CD4 count ≥200 cells/mm^3^, as well as all sero-negative mothers will be randomised to either CTX daily or SP IPT. After all inclusion and exclusion criteria are met, study explained and women are willing to participate in the study and sign the Informed Consent. She will be randomized to one treatment and get the next available study-ID number which has a pre-established treatment allocation. The randomisation will be stratified within each group. HIV-infected pregnant women with CD4 count <200 cells/mm^3^ will not be included in the RCT as they will be assigned to only one intervention, i.e. CTX daily according to the current national guidelines. Women CD4 count below 200/µL will receive anti-retroviral treatment (ART). The ART given to adults and adolescents in Zambia is as follows: Truvada with either Nevirapine or Efavirenz (Truvada is a combination of Tenofovir (TDF) and Emtricitabine (FTC). In pregnancy, Truvada is given with Nevirapine mainly.

HIV infected women with CD4<200/µL, will be included as an observational cohort.

Randomization will be performed in blocks of 10. A randomization list will be generated by the CTU unit using tables of random numbers and will be used to number a sequence of envelopes that will contain the regimen assignments for each pregnant woman in the trial.

### Treating the patient

This being a chemoprophylactic study, all pregnant women randomised to the CTX group will be treated with 2 tablets of co-trimoxazole single-strength tablets (400mg of sulphamethoxazole and 80mg of trimethoprim) to be taken daily. These will be requested to routinely visit the ANC monthly to ensure adherence.

Those pregnant mothers randomised to the SP group shall be provided with SP to be taken as per routine ANC policy and as per WHO guidelines. All women will be scheduled to receive three doses of SP for the prevention of malaria. They will be given a month supply of 200 mg of ferrous sulphate, 5mg of folic acid and one dose of a deworming medication. They will be asked not to self-administer antimalarials in addition to the study medication. They will be asked to report to the study site for evaluation if they experience symptoms of malaria at any time between scheduled study visits

## Adverse Events

### Definition of an adverse event

An AE is any untoward medical occurrence in a patient or clinical investigation subject administered a pharmaceutical product and which does not necessarily have a causal relationship with this treatment.

An AE can therefore be any unfavourable and unintended sign (that could include a clinically significant abnormal laboratory finding), symptom or disease temporally associated with the use of a medicinal product, whether or not considered related to the medicinal product.

### Severity, relationship of event to study drug, and outcome

The severity of a clinical adverse event is to be scored according to the following scale:

1 Mild Awareness of sign or symptom, but easily tolerated

2 Moderate Discomfort enough to cause interference with usual activity

3 Severe Incapacitating with inability to work or perform usual activity

4 Life-threatening Patients at risk of death at the time of the event

The relationship of an adverse event to study drug is to be assessed according to the following definitions:

1 Definitely unrelated

It should be reserved for those events which occur prior to test drug administration (e.g., washout or single-blind placebo) or for those events which cannot be even remotely related to study participation (e.g. injury caused by a third party).

2 Unlikely

There is no reasonable temporal association between the study drug and the suspected event and the event could have been produced by the subject's clinical state or other modes of therapy administered to the subject.

3 Possible

The suspected adverse event may or may not follow a reasonable temporal sequence from study drug administration but seems to be the type of reaction that cannot be dismissed as unlikely. The event could have been produced or mimicked by the subject's clinical state or by other modes of therapy concomitantly administered to the subject.

4 Likely

The suspected adverse event follows a reasonable temporal sequence from study drug administration, abates upon discontinuation of the drug, and cannot be reasonably explained by the known characteristics of the subject's clinical state.

5 Definitely related

It should be reserved for those events which have no uncertainty in their relationship to test drug administration: this means that a re-challenge was positive.

The outcome of each AE must be assessed according to the following classification:

| - Completely recovered : | The patient has fully recovered with no observable residual effects |
| --- | --- |
| - Not yet completely recovered : | Improvement in the patient’s condition has occurred, but the patient still has some residual effects |
| - Deterioration : | The patient’s overall condition has worsened |
| - Permanent damage : | The AE has resulted in a permanent impairment |
| - Death : | The patient died due to the AE |
| - Ongoing : | The AE has not resolved and remains the same as at onset |
| - Unknown : | The outcome of the AE is not known because the patient did not return for follow-up (lost to follow-up) |

### Definition of a serious adverse event

A serious adverse event (experience) (SAE) or reaction is any untoward medical occurrence that at any dose fulfils at least one of the following criteria:

- results in death;
- is life-threatening;
- requires hospitalization or prolongation of existing hospitalization ( except for indications that are non-medical such as delivery or observation pending delivery due to geographical distance);
- results in persistent or significant disability/incapacity;
- requires a specific medical or surgical intervention to prevent one of the outcomes listed above;

All reported adverse events will be documented in the case report form and classified as mild, moderate or severe. All serious adverse events (SAEs) will be reported to the PI within 24 hours (working day) of being notified of the event. If the AE was considered likely to be associated with the study medication, this patient will be discontinued from further study procedures. Therefore, the study drug shall be discontinued in case of any serious adverse reaction for which causality of the event shall be strongly related.

### Follow-up ANC procedures

Women will be followed according to the standard obstetrical schedule. Mothers will be offered assistance with transportation to the health care facility for routine visits as well as delivery in form of transport refunds. The mothers will be seen on a monthly basis at the participating health centers. At each follow-up visit, the study subjects will be asked if they have had any malaria symptoms since their last visit and whether they have taken any antimalarial medication from public, private, or traditional sectors. They will be asked if they have taken iron supplements on a regular basis since their last visit. Temperature and blood pressure will be performed at each visit. Hb will be measured once early in the third trimester, between weeks 30-34. They will also be asked about any possible drug reactions to the study medication. Further, doses of the study medications will be withheld if the woman reports serious skin or systemic reactions thought to be related to the study drugs. Each mother’s supply of SP or CTX will be kept at the health center.

At each monthly visit, either SP (three tablets from the randomization envelop) or CTX (2 tablets/day) will be administered under directly observed therapy. Women in the CTX group will be given the drug supply for the whole month. Mothers presenting between 16 and 24 weeks of gestation will be given their second doses of SP from the randomization envelop at their 3^rd^ follow-up visit (i.e., 12 weeks after initial evaluation). Mothers who begin the study on weeks 25 or 26 of gestation will be administered their second SP doses at their 2^nd^ follow-up visit. No doses of SP will be administered later than week 36 of gestation. Although pregnant women living with HIV widely use co-trimoxazole, there is no evidence of an increase in co-trimoxazole-related adverse events among pregnant women versus non-pregnant women ([[47]](#endnote-48)).

If a mother does not show for her monthly visit, community health workers (CHW) or any other designated study staff will make a home visit within 3 days of the scheduled visit. The CHW will encourage the woman to take her prescribed prophylaxis and also keep her next ANC clinic appointment.

### Breakthrough malaria episodes

Any pregnant woman following the study shall be treated for any malaria episode that occurs during the period of follow-up using the standard treatment guidelines prevailing for malaria drug treatment policy of pregnant women in Zambia. Owing to the fact that women will be receiving SP as part of the protocol, symptomatic malaria will be managed with the current second line drug, quinine, dosed at 600 mg every 8 hours over a 5-day period or as per decision of the attending clinician. Women with symptomatic malaria will have blood taken by finger prick and then will have Hb measured in the clinic. Treatment with quinine or any other medication will be documented in the case report form. Treated women will be asked to return to the ANC within a week for re-evaluation. If the attack of malaria has resolved, then she will continue with her next scheduled study visit. If a woman is suspected to have severe malaria or is found to have severe anaemia, she will be referred to hospital staff for care.

### Concomitant treatment

Women who receive subsequent treatment with any drug for any condition that may arise during the follow-up period will continue with the study. The subsequent drug exposures shall be documented along with the conditions for which they were received.

### Patient withdrawal/ discontinuation

The study participants will be free to withdraw from the study at any time, without necessarily giving reasons as the study shall be conducted under ICH/GCP guidelines.

Following are reasons for discontinuing a participant from the study:

- Withdrawal of consent
- Movement from the catchment area
- Development of serious adverse events that are considered probably or definitely due to study drug (SP or CTX)
- Development of eclampsia or gestational diabetes
- Unavailability for study procedures for ≥3 consecutive scheduled visits

The study participants may also be lost to follow-up for any reason. However, the Patient record Forms (PRF) for all enrolled patients, including those prematurely discontinued from the study, will be collected for data entry.

## Delivery procedures

At delivery, the status of the baby will be assessed, with regard to vital signs, birth weight, gestational age. Gestational age will be confirmed using the Dubowitz neurological and external criteria for gestational assessment at birth. Staff at the Choma sites has gained substantial experience in performing the Dubowitz assessment during the Coartem® pregnancy registry. Major and minor birth defects detection will be performed at birth according to a pre-determined checklist. The maternal vital status would be established and recorded at delivery and at 6 weeks after delivery. Placental tissue and maternal blood plus filter paper will be collected at delivery.

Incentives, a mother’s pack (items ordinarily purchased by women for health center delivery) and transportation reimbursement will be provided to mothers who deliver at the ANC where they received their pre-natal care. At delivery, study participants will be asked about malaria symptoms, treatment for malaria, and any pregnancy complications since the last study visit.

Newborns will be weighed on a digital scale, accurate to the nearest gram, within 24 hours of delivery. Gestational age will be determined by physical and neurologic examination using a modified standardized Dubowitz method ([[48]](#endnote-49)).

### PMTCT

Zambia guidelines for prevention of mother to child transmission and also mainly used in Choma district are the two regimens:

The two-dose NVP regimen found in the Uganda HIVNET 012 protocol to reduce perinatal transmission by 50% ([[49]](#endnote-50)) will be offered as a service to all women who consent to VCT and their infants at the two sites. The regimen consists of a single 200 mg tablet of NVP (Boehringer-Ingelheim Pharmaceuticals) given to the mother at the onset of labor, and a suspension of 2mg/kg given to the neonate before discharge (or within 72 hours of birth).

All HIV-infected participants will be offered nevirapine. They will be given nevirapine during post-test counseling and will be instructed to take it at onset of labor. These instructions will be reiterated at all of the follow-up ANC visits. When arriving at the clinic for delivery, HIV-infected participants who accepted nevirapine will be asked if they took the nevirapine pill, and this will be recorded. If the pill was not taken, the participant will be given a new nevirapine pill to be taken immediately.

If a participant is dosed in false labor, she will receive an additional dose at onset of active labor if more than 24 hours have passed since initial dosing. Participants who vomit within 30 minutes of dosing will be re-dosed; participants who vomit more than 30 minutes after dosing with nevirapine will not be re-dosed.

AZT 300mg tablets twice daily from 34 weeks of pregnancy and 300mg orally every three hours during delivery.

#### Maternal dosing:

For spontaneous labor, the mother should be assessed as soon as possible after arriving in labour and delivery. If she has not yet taken 200 mg NVP at onset of labor, dosing should proceed when in the clinician's best judgment the woman is expected to deliver within 24 hours of presentation. A second dose of 200 mg NVP will be given if the woman remains in labor for 48 hours or more.

If a woman has taken NVP and is subsequently assessed to be in false labor, she should re-initiate study dosing procedures as outlined above if she returns in labor and more than 48 hours have passed since she received NVP.

For an emergency or non‑elective Cesarean section, the woman should receive NVP as soon as the decision is made to do the Cesarean section.

For an elective Caesarean section, the NVP will be given 4-12 hours before the scheduled procedure. Women who vomit within 30 minutes of dosing will receive a second 200 mg dose of NVP. Women who vomit more than 30 minutes after dosing will not receive an additional dose.

#### Infant dosing:

The newborn will be given the oral NVP, as soon as possible after delivery but **within 72 hours** post birth. Should the infant vomit within 30 minutes of NVP administration, the dose will be re-administered once.

### Placental smears and histology

At the time of delivery, a thick smear will be taken from the maternal surface of the placenta. The thick smear will be stained with Giemsa and then examined for parasites and malaria pigment. A placental biopsy specimen will also be taken for histological examination. A 1 cm^3^ biopsy will be taken from the maternal side of the placenta approximately one fourth of the distance from the centre to the edge of the placenta ([[50]](#endnote-51)). Previous studies have demonstrated that placental pathology in malaria infection is greatest in the medial and lower placental zones([[51]](#endnote-52)). The biopsy will be immediately placed in 20 mL neutralized formal-saline and will be sent to Tropical Diseases Research Centre, then to Ndola Central Hospital Pathology Department for histologic examination within one month (as described in the Procedure Manual). A histopathologist shall be responsible the examination of the placentas and for all GCP compliant procedures related to this essential component of the study. External quality control shall be linked to the Institute of Tropical Medicine in Antwerp, Belgium.

### Definitions of placental malaria

1. **Acute infection**. The following three findings must be present: parasitized erythrocytes, malaria pigment in intervillous erythrocytes, and intervillous monocyte/macrophage infiltrates containing malaria pigment. The presence of malaria pigment in the intervillous neutrophils and pigment in fibrin are not necessary for this definition.

2**. Recent infection**. No parasites should be observed but malaria pigment in intervillous erythrocytes and in intervillous monocyte/macrophage infiltrates must be present. There may also be pigment in the intervillous neutrophils and pigment in fibrin.

3. **Past infection.** There must be only malaria pigment present in fibrin but no parasites and no pigment in intervillous erythrocytes or monocyte/macrophage infiltrates.

4. **Uninfected.** There must not be any parasites or malaria pigment in fibrin or any blood cells.

## Mother and infant follow-up procedures

The infant will be assessed for adverse outcomes associated with increased SP dosing, twice after delivery, once at 7 days of age, and again at 6 weeks. The general health status of the infant, including an examination for scleral icterus will be performed on all neonates at 7 days and 6 weeks of life. Infants who are not brought to the clinic for follow-up visits will be sought at home within 3 days of the scheduled visit. Any infant showing signs of illness will be referred to a hospital or clinic for evaluation.

Mothers will be seen 6 weeks after delivery and will be asked about stillbirths (if the delivery is not recorded at the study site) and neonatal deaths. If a woman does not return for this follow-up visit, then they will be sought at their home.

The final visit will take place 6 weeks after delivery, the maternal status and the status of the baby will be established. Unfortunately for the mothers who would have delivered at home placental parasitaemia would have not been possible to establish at birth.

# Safety monitoring

During each ANC visit, the study participant will be asked if she has had any adverse events. All reported adverse events will be documented in the case report form and classified as mild, moderate or severe.. All serious adverse events will be reported to the IEC (Independent Ethical Committee) of the University of Antwerp and the TDRC and the IRB of the ITM for information and comments on the ethical aspects of the event. The SAE will be reported within 24 hours (working day) of being notified of the event by the site study team. If the SAE was considered likely to be associated with the study medication, this patient will be discontinued from further study procedures

## Data Safety Monitoring Board (DSMB)

We shall not constitute an independent data safety monitoring board as SP and CTX are well known and approved standard drug in Pregnancy (40,[[52]](#endnote-53)).

# Case Report Form (CRF)

## Presentation of the CRF

All data and observations must be initially documented in the source documents (Patient Record Form - PRF), and then entered in the Case Report Form.

For this study, an electronic CRF will be used, which can be defined and entered remotely using the Internet, or entered offline on a laptop and synchronised with the main database at a later time. The e-CRF will allow identification of the study, site and patient; recording of the selection and inclusion of patients in the study; recording of all data collected at each different study visits; recording of possible adverse events and any suspension of the study.

The Data Handling Protocol will describe in detail the design of the e-CRF and of the database; the remote and/or offline data entry; the list of validation; the methods of import/export of data; the details of how import/export of data are validated; and the data access privileges.

## Study protocol and related documentation

All study related documents including the study protocol, manuals of operations; all correspondence sent to or received from the study monitor; materials used for obtaining informed consent; protocol modifications and records of the local Ethical Committee approval; and all communications with the local Ethics Committee will be maintained at the study site as well.

## Record retention

Retention of accurate and complete records is essential to establish the validity and completeness of the study. All source documents will be retained for 10 to 15 years at the TDRC archive (GCP compliant) after the data has been published.

# Monitoring and quality assurance

The task of the Monitor (to be identified by the sponsor) is to verify the best conduct of the study through frequent contacts by phone and in person with the Principal Investigator and site staff, in accordance with the study-specific Standard Operating Procedures and with Good Clinical Practice requirements, with the purposes of facilitating the work and attaining the objectives of the study. These visits will enable the Monitor to maintain current, personal knowledge of the study through review of the records, comparison with source documents, observation and discussion of the conduct of the study with the Investigator.

Each site will be visited 3 times during the conduct of the trial plus a study initiation visit at the start of clinical activities and a close-out visit after the last patient has completed the follow up. The monitor will carry out at least 30% source data verification.

The Choma site investigator must maintain source documents for each patient in the study, consisting in case and visit notes (hospital or clinic medical records) containing demographic and medical information, laboratory data, and the results of any other tests or assessments. All information in the electronic CRF must be traceable to these source documents in the patient’s file. The investigator must keep the original informed consent form signed by the patient’s parent or guardian and send a copy to the sponsor. The investigator must give the monitor access to all relevant source documents to confirm their consistency with the electronic CRF entries.

The Principal Investigator from TDRC and each co-Investigator by signing this protocol declare that he/she will permit trial-related monitoring, audits, Independent Ethic Committee review, and regulatory inspections, providing direct access to source data/documents.

The Principal Investigator and co-Investigators agree to conduct the present study in full agreement with the principles of the “Declaration of Helsinki” and subsequent relevant amendments (see Appendix III).

# Data Management

## Data Handling

Clinical data, as requested in the protocol, will be collected and recorded in the appropriate e-CRF. Laboratory data, as requested in the protocol will be registered at the laboratory and entered in the e-CRF. All data will then be processed from the e-CRF into a GCP-compliant Clinical Data Management System, which will be described in details in a Data Handling Protocol.

During the conduct of the study data will be verified and reviewed to produce and maintain high quality data. The Data Handling Protocol will detail the validation checks that can be incorporated into the electronic forms and the audit trail which is maintained during all data entry and editing of records. All unresolved issues will be queried and resolved before locking the database. Data transfer and handling is done with appropriate security measures and with regard to rights, safety and well-being of trial subjects.

A report on data management process will be produced. The report will include

- A full field listing and description of the file structure of the electronic data
- Reference ranges and units for laboratory data
- A list a brief description of all programs run on the data
- Level of errors found at each stage of checking the data
- General comments on data quality and significant problems encountered with the data
- A detailed list of any unresolved data queries
- A statement of any queries/errors which have not been corrected on the database
- A statement of the storage location of the electronic database

The statistical team (CTU) will review the database prior to finalisation. They will report on any problem encountered during the analysis. Any changes to the database after the lock can only be made by joint written agreement between the statistical team, the data manager and the study coordinator.

## Data analysis plan

The efficacy of SP and CTX for the prevention of placental malaria and maternal peripheral parasitemia will be analyzed on an intent-to-treat basis. Participating women who have placental and peripheral blood smears done at the time of delivery will be included in analyses of placental parasitemia and maternal peripheral parasitemia. Live singleton neonates who have birth weights taken at the time of delivery will be included in the analysis of birth weight. Results from all women who deliver singleton neonates will be included in birth outcome and neonatal survival analyses. The inclusion of multiple pregnancies at data analysis, affects the interpretation of the secondary outcomes mentioned due to the resultant birth weights and possible consequent neonatal survival from them. However, multiple pregnancy is not an exclusion criterion.

Differences between means will be tested using a student’s t test, once the normality of the outcomes is established. Differences between proportions will be evaluated using the Chi-square test. Demographic, disease stage or other parameters which are potential predictors or covariates that are found to be significant at the 0.10 level will be entered in logistic regression equations in a step-wise manner. Should a significant decrease in any of the primary or secondary outcomes be associated with the study treatment, we will use multivariate modelling to investigate if significant covariates exist.

To assess whether important risk factors for the primary event of interest are differentially distributed among the two study prophylactic options, frequency distributions of characteristics of women in the two groups will be described, Key characteristics include timing of breakthrough malaria episodes, socioeconomic status, smoking status, alcohol abuse during pregnancy, concomitant infections, concomitant medications and their timing and maternal anaemia and concomitant diseases/ infections and their timing.

## Outcome Definitions

### Primary outcomes

- Placental malaria infection will be defined as the presence of placental malaria either acute, recent or past infection as defined by the histopathological examination. (see 13.5.2.)

### Secondary outcomes

- Acute placental malaria infection will be defined by the hystopathological examination (see 13.5.2.).
- Recent placental malaria infection will be defined by the hystopathological examination (see 13.5.2.)
- Past placental malaria infection will be defined by the hystopathological examination Maternal peripheral parasitemia will be defined as the presence of asexual stage parasites in thick smears made from maternal peripheral venous blood (see 13.5.2.)
- LBW = birth weight <2,500 grams
- Premature delivery = delivery prior to 37 weeks gestation
- Spontaneous miscarriage = any spontaneous abortion before the end of gestation
- Stillbirth
- Cord blood parasitemia = presence of asexual stage parasites in thick smears
- Neonatal death = infant death within the first 28 days of life
- Maternal anemia = Hb <11.0 g/dL
- Maternal severe anemia = Hb <6 g/dL
- Symptomatic maternal malaria infection = axillary temperature ≥37.5^o^C and asexual parasitemia
- Severe maternal adverse reactions to SP = severe cutaneous reactions (e.g., erythema multiforme, Stevens-Johnson syndrome, or toxic epidermal necrolysis)

# Limitations

The unsupervised intake of daily CTX remains a challenge in relation to compliance especially for non HIV pregnant women.

There is substantial evidence that malaria infection can increase the risk of stillbirth, perinatal mortality, low birth weight, gestational age and maternal mortality. Therefore, the occurrence of malaria should be well documented and accounted for in analyses.

Although many efforts are planned to minimize loss to follow-up, the study may remain limited in its capacity to track spontaneous abortion cases, birth defects and still births in home deliveries.

# Timeline

We estimate to start in October, 2008. In 10 months time we will be able to recruit the required number of patients. Data will be analyzed in 2010. Final results for publication should be available by September 2010.

|  | 2008 | | | | 2009 | | | | 2010 | | | |
| --- | --- | --- | --- | --- | --- | --- | --- | --- | --- | --- | --- | --- |
| Preparation, Training |  |  |  |  |  |  |  |  |  |  |  |  |
| Enrolment |  |  |  |  |  |  |  |  |  |  |  |  |
| Intervention |  |  |  |  |  |  |  |  |  |  |  |  |
| Laboratory work |  |  |  |  |  |  |  |  |  |  |  |  |
| Data entry, cleaning and analyses |  |  |  |  |  |  |  |  |  |  |  |  |
| Information to health authorities |  |  |  |  |  |  |  |  |  |  |  |  |

# Ethical considerations

## Study design

This Randomized Clinical Trial (RCT) is limited to HIV negative and HIV infected with CD4 count equal or above 200/µL. Indeed, as the benefits of CTX for maternal health are already proven. Ethical constraints limit the possibility of conducting randomized trials to test the possible effects that interventions, such as cotrimoxazole, have on neonatal outcomes in immunosupressed pregnant women. One of the few ways to investigate the effects of cotrimoxazole in this group is to use historical controls. A constraint to this approach is that malaria endemicity is variable and changing rapidly and data are not always available.

Therefore, to assess the impact of CTX on malaria in pregnancy in immune suppressed HIV infected pregnant women, we‘ve chosen the following strategy: immune suppressed women (or women already on CTX/ARV treatment) eligible for all other study criteria will be prospectively followed up while being on ARV/CTX treatment. On the other hand, all patients who solely report to the health facility at time of delivery will follow the normal procedures (including VCT); Women who turn out to be HIV positive will receive PMTCT as stipulated in the Zambian guidelines. If HIV infected women have a <200 CD4 count/mm^3^ and there are on co-trimoxazole prophylaxis or ARV treatment given, they shall be requested to provide informed consent to have an interview using a structured questionnaire to assess how many malaria attacks, several other potential risk factors they may have had in the current pregnancy, and factors to assess selection bias

## Informed consent procedures

All women eligible will receive detailed information on the study. They will be asked if they, are willing to follow all procedures including coming to that health centre for delivery. Informed consent form will be signed. If the woman is illiterate, a witness (not connected with the research study) will sign the consent, attesting to the fact that the consent was accurately read or interpreted. If the a legal guardian is available, he/she will be asked come to ANC where he/she will be informed about the study. Women will be receiving antimalarials that have been widely used and are generally known to be safe.

## Confidentiality

Questionnaires and consent forms to be used will be included in the Annexes to this document. Confidentiality will be maintained to the extent allowed by law, except when being used for study participant follow up. Participant clinical forms will be kept in a locked cabinet and will be available only to the study coordinator.

## Ethical review

This protocol and data collection instruments will be reviewed and approved by the ethical and scientific committee of the University of Antwerp and the Tropical disease Research Centre in Ndola, Zambia

## Potential risks

While SP is a safe and standard treatment for malaria, women who participate in this study will be subject to the risk of a sulfa allergic reaction to the course of treatment. SP has been widely used as an antimalarial and is known to be efficacious in clearing peripheral and placental parasitaemia in Zambia and other African countries.. Sulfa drugs carry a known but low risk for severe adverse side effects. Based on a study carried out among North American travelers, serious side effects of SP and other sulfa drugs are rare but do include severe cutaneous reactions (erythema multiforme, Stevens-Johnson syndrome, and toxic epidermal necrolysis), which occur in approximately 1 per 5,000-8,000 users, with fatal reactions occurring in an estimated 1 per 11,000-20,000 users. Women will be informed of this risk prior to participation. They will be informed to return to ANC should they experience any possible adverse reaction after receiving SP. In addition, any woman reporting a history of allergic reactions to sulfa drugs and other unknown medications will be excluded from the study.

Sulfa drugs have been widely used during pregnancy for the treatment of asymptomatic bacteriuria and cystourethritis, as well as for toxoplasmosis. They have also been used in malaria prophylaxis and treatment trials, which included pregnant women, without adverse pregnancy effects. The most common concern expressed in the medical literature regarding the use of SP during pregnancy is a theoretical one that its use in late pregnancy may displace bilirubin from albumin in the neonate and thus contribute to kernicterus. This concern stems from one study that demonstrated an increased incidence of kernicterus in neonates treated with a sulfonamide, in combination with several other drugs, but has not been confirmed in subsequent studies. Recently, a study found an association with the use of folic acid antagonists during pregnancy and cardiovascular defects and cleft palates. However, this study failed to show a significant increase in risk when the folic acid antagonist was taken in the second or third trimester of pregnancy. Since the original report, there has been only one report of 9 infants with hyperbilirubinemia, including one who had kernicterus after administration of sulfa drugs to the mother. Other trials have failed to show adverse reactions in neonates born to mothers taking sulfonamides during pregnancy, including studies of women who received sulfa drugs throughout pregnancy.

In the a trial in pregnant women in Kenya, there was no difference in the incidence of neonatal jaundice among treatment groups, 2-dose SP, monthly SP, and a case management group. Many women in the case management group received no SP during pregnancy. Despite this unproven risk of neonatal kernicterus in women who have taken sulfa drugs in late pregnancy, an effort will be made in the current protocol to ensure that SP is not given in the last month of pregnancy. Before treatment, current gestational age for the pregnancy will be determined. Although there is little evidence that sulfonamides are teratogenic, so as to minimize any drug exposure during embryogenesis, drug use will be avoided in the first trimester of pregnancy. In an area with high transmission of malaria and high incidence of LBW, the risk of malaria-associated adverse outcomes outweighs the risk of adverse reaction to sulfa.

Dosing with SP will be no more frequent than every 30 days and women will be monitored for the occurrence of a rash, which if it develops will be treated symptomatically. A recent review reconfirmed, sulfadoxine/pyrimethamine - when delivered as IPTp - have a favourable safety profile (40, Error: Reference source not found).

## Anticipated benefits

Benefits to participating women include treatment of malarial illness, supplementation with iron and folate during their pregnancies (ensuring these are available as they sometimes run out in routine ANCs), medical assistance throughout the pregnancy and during delivery. All patients will be invited to submit blood for HIV testing. For those mothers who are HIV negative, this will give them an opportunity to know their status and to learn about methods to prevent acquiring HIV. For those pregnant mothers who are HIV positive, their infants will benefit from intervention with Niverapine to decrease the risk of mother-to-child transmission.

## Confidentiality issues

Each study participant will be assigned a unique study number and will be issued a study ID card with the same number. The baseline visit encounter form includes the patient’s name and contact information on a face page. This page also includes the study ID number. This face page will be kept separately from other files in a locked file, accessible only by the study PI, or her designate at times when she is physically absent from the study site. All subsequent encounter forms will be linked only by study ID number and will have no personal identifying information. The face page information can be accessed only with the approval of the PI or temporary designate and with the purpose of tracking women who have missed appointments to provide participants with urgent information that may emerge during the study. All field study personnel with access to identifiable information will be required to sign a confidentiality agreement in order to guarantee the security of information pertaining to individual patients. The study number will identify all forms pertaining to the results of interviews, the clinical examinations, and the laboratory tests. Computer datasets generated from such forms will also lack identifiable data. Linkage of results and identifiable data will be done only when necessary by authorized study personnel.

# Appendices

Appendix 1: Dubowitz Test for Gestational Age Assessment

Appendix 2: Major and Minor Birth Defects Recording

## Appendix 1: Dubowitz Test for Gestational Age Assessment

| GESTATIONAL AGE ASSESSMENT   |  | | --- | |  |  |  |  |  | |  | |  |  |  |  |  | |  |  | |  |  | |  | | |  | |  | |  | | |  | | **ID No** | | | | | | | |  | | |  | | |  | |  |  |  |  |  |  |  | |  |  | |  |  |  | | | | |  | |  |  |  | |  |
| --- | --- | --- | --- | --- | --- | --- | --- | --- | --- | --- | --- | --- | --- | --- | --- | --- | --- | --- | --- | --- | --- | --- | --- | --- | --- | --- | --- | --- | --- | --- | --- | --- | --- | --- | --- | --- | --- | --- | --- | --- | --- | --- | --- | --- | --- | --- | --- | --- | --- | --- | --- | --- | --- | --- | --- | --- | --- | --- | --- | --- | --- | --- | --- | --- | --- | --- | --- | --- | --- | --- | --- | --- | --- |
|  |  |  |  |  |  | |  | |  |  |  |  |  | |  |  | |  |  | |  | | |  | |  | |  | | |  | | **DOB** | | | | | | | |  | | |  | | |  | |  |  |  |  |  |  |  | |  |  | |  |  | **AGE** | | | | |  | |  |  | **EXAMINER** | | |
|  |  |  |  |  |  | |  | |  |  |  |  |  | |  |  | |  |  | |  | | |  | |  | |  | | |  | | **DOE** | | | | | | | |  | | |  | | |  | |  |  |  |  |  |  |  | |  |  | |  |  | **SEX** | | | | |  | |  |  | **Race** | | |
| **NEUROLOGICAL CRITERIA** | | | | | | | | | | | | | | | | | | | |  | | |  | |  | |  | | |  | |  | | |  | | | |  | | |  | |  | | |  | |  |  |  |  |  |  |  | |  |  | |  |  |  |  | |  | | |  |  |  |  | | |
| NEURO SIGN | | | | | | SCORE | | | | | | | | | | | | | | | | | | | | | | | | | | | | | | | | | | | | | | | | | | | | | | | | | | | | | | | | | | RECORD SCORE HERE | | | | | | | **BIRTH WEIGHT** | | |
|  |  |  |  |  |  | 0 | | | | | | | | 1 | | | | | | | | 2 | | | | | | | | | | | | | | | 3 | | | | | | | | | | | | | | 4 | | | | | | 5 | | | | | | |  |  |  |  |  |  |  |  | |  |
| POSTURE | | | | | | |  | | --- | | | | | | | | | | |  | | --- | | | | | | | | | | |  | | --- | | | | | | | | | | | | | | | | | |  | | --- | | | | | | | | | | | | | | | | |  | | --- | | | | | | | |  | | | | | | |  | | | | | | |  | |  |
|  |  |  |  |  |  |  |  |  |  |  |  |  |  |  |  |  |  |  |  |  |  |  |  |  |  |  |  |  |  |  |  |  |  |  |  |  |  |  |  |  |  |  |  |  |  |  |  |  |  |  |  |  |  |  |  |  |  |  |  |  |  |  |  |  |  |  |  |  |  |  | **LENGTH** | |  |
| SQUARE WINDOW | | | | | | | 90 | | --- | | | | | | | | | | | 60 | | --- | | | | | | | | | | | 45 | | --- | | | | | | | | | | | | | | | | | | 30 | | --- | | | | | | | | | | | | | | | | **0** | | | | | |  | | | | | | |  | | | | | | |  | |  |
|  |  |  |  |  |  |  |  |  |  |  |  |  |  |  |  |  |  |  |  |  |  |  |  |  |  |  |  |  |  |  |  |  |  |  |  |  |  |  |  |  |  |  |  |  |  |  |  |  |  |  |  |  |  |  |  |  |  |  |  |  |  |  |  |  |  |  |  |  |  |  | **HEAD CIRCUMFERENCE** | | |
| ANKLE DORSIFLEXION | | | | | | | 90 | | --- | | | | | | | | | | | 75 | | --- | | | | | | | | | | | 45 | | --- | | | | | | | | | | | | | | | | | | 20 | | --- | | | | | | | | | | | | | | | | | **0** | | --- | | | | | | | |  | | | | | | |  | | | | | | |  | |  |
|  |  |  |  |  |  |  |  |  |  |  |  |  |  |  |  |  |  |  |  |  |  |  |  |  |  |  |  |  |  |  |  |  |  |  |  |  |  |  |  |  |  |  |  |  |  |  |  |  |  |  |  |  |  |  |  |  |  |  |  |  |  |  |  |  |  |  |  |  |  |  | **ARM CIRCUMFERENCE** | | |
| ARM RECOIL | | | | | | | 180 | | --- | | | | | | | | | | | 90-180 | | --- | | | | | | | | | | | <90 | | --- | | | | | | | | | | | | | | | | |  | | | | | | | | | | | | | |  | | | | | |  | | | | | | |  | | | | | | |  | |  |
|  |  |  |  |  |  |  |  |  |  |  |  |  |  |  |  |  |  |  |  |  |  |  |  |  |  |  |  |  |  |  |  |  |  |  |  |  |  |  |  |  |  |  |  |  |  |  |  |  |  |  |  |  |  |  |  |  |  |  |  |  |  |  |  |  |  |  |  |  |  |  |  |  |  |
| LEG RECOIL | | | | | | | 180 | | --- | | | | | | | | | | | 90-180 | | --- | | | | | | | | | | | <90 | | --- | | | | | | | | | | | | | | | | |  | | | | | | | | | | | | | |  | | | | | |  | | | | | | |  | | | | | | |  | |  |
|  |  |  |  |  |  |  |  |  |  |  |  |  |  |  |  |  |  |  |  |  |  |  |  |  |  |  |  |  |  |  |  |  |  |  |  |  |  |  |  |  |  |  |  |  |  |  |  |  |  |  |  |  |  |  |  |  |  |  |  |  |  |  |  |  |  |  |  |  |  |  | **SCORE** | | **GA** |
| POPLITEAL ANGLE | | | | | | | 180 | | --- | | | | | | | | | | | 160 | | --- | | | | | | | | | | | 130 | | --- | | | | | | | | | | | | | | | | | | 110 | | --- | | | | | | | | | | | | | | | | | 90 | | --- | | | | | | | | | <90 | | --- | | | | | | | | |  | | | | | | | 10 | | 27.2 |
|  |  |  |  |  |  |  |  |  |  |  |  |  |  |  |  |  |  |  |  |  |  |  |  |  |  |  |  |  |  |  |  |  |  |  |  |  |  |  |  |  |  |  |  |  |  |  |  |  |  |  |  |  |  |  |  |  |  |  |  |  |  |  |  |  |  |  |  |  |  |  | 12 | | 27.8 |
| HEEL TO EAR | | | | | | |  | | --- | | | | | | | | | | |  | | --- | | | | | | | | | | |  | | --- | | | | | | | | | | | | | | | | | |  | | --- | | | | | | | | | | | | | | | | |  | | --- | | | | | | | |  | | | | | | |  | | | | | | | 14 | | 28.3 |
|  |  |  |  |  |  |  |  |  |  |  |  |  |  |  |  |  |  |  |  |  |  |  |  |  |  |  |  |  |  |  |  |  |  |  |  |  |  |  |  |  |  |  |  |  |  |  |  |  |  |  |  |  |  |  |  |  |  |  |  |  |  |  |  |  |  |  |  |  |  |  | 16 | | 28.8 |
| SCARF SIGN | | | | | | |  | | --- | | | | | | | | | | |  | | --- | | | | | | | | | | |  | | --- | | | | | | | | | | | | | | | | | |  | | --- | | | | | | | | | | | | | | | |  | | | | | |  | | | | | | |  | | | | | | | 18 | | 29.4 |
|  |  |  |  |  |  |  |  |  |  |  |  |  |  |  |  |  |  |  |  |  |  |  |  |  |  |  |  |  |  |  |  |  |  |  |  |  |  |  |  |  |  |  |  |  |  |  |  |  |  |  |  |  |  |  |  |  |  |  |  |  |  |  |  |  |  |  |  |  |  |  | 20 | | 29.9 |
| HEAD LAG | | | | | | |  | | --- | | | | | | | | | | |  | | --- | | | | | | | | | | |  | | --- | | | | | | | | | | | | | | | | | |  | | --- | | | | | | | | | | | | | | | |  | | | | | |  | | | | | | |  | | | | | | | 22 | | 30.4 |
|  |  |  |  |  |  |  |  |  |  |  |  |  |  |  |  |  |  |  |  |  |  |  |  |  |  |  |  |  |  |  |  |  |  |  |  |  |  |  |  |  |  |  |  |  |  |  |  |  |  |  |  |  |  |  |  |  |  |  |  |  |  |  |  |  |  |  |  |  |  |  | 24 | | 30.9 |
| VENTRAL SUSPENSION | | | | | | |  | | --- | | | | | | | | | | |  | | --- | | | | | | | | | | |  | | --- | | | | | | | | | | | | | | | | | |  | | --- | | | | | | | | | | | | | | | | |  | | --- | | | | | | | |  | | | | | | |  | | | | | | | 26 | | 31.5 |
|  |  |  |  |  |  |  |  |  |  |  |  |  |  |  |  |  |  |  |  |  |  |  |  |  |  |  |  |  |  |  |  |  |  |  |  |  |  |  |  |  |  |  |  |  |  |  |  |  |  |  |  |  |  |  |  |  |  |  |  |  |  |  |  |  |  |  |  |  |  |  | 28 | | 32.0 |
|  |  |  |  |  |  | |  | |  |  |  |  |  | |  |  | |  |  | |  | | |  | |  | |  | | |  | |  | | |  | | | | **TOTAL NEUROLOGICAL** | | | | | | | | | | | | | | | | |  |  | |  |  |  |  | |  | | | | | | 30 | | 32.5 |
|  |  |  |  |  |  | |  | |  |  |  |  |  | |  |  | |  |  | |  | | |  | |  | |  | | |  | |  | | |  | | | |  | | |  | |  | |  | |  |  |  |  |  |  |  | |  |  | |  |  |  |  | |  |  |  |  |  |  | 32 | | 33.0 |
| **EXTERNAL (SUPERFICIAL) CRITERIA** | | | | | | | | | | | | | | | | | | | | | | | | | | | | | | | | | |  | | | |  | | |  | | |  | | |  | |  |  |  |  |  |  |  | |  |  | |  |  |  |  | |  | | |  |  |  | 34 | | 33.6 |
| EXTERNAL SIGN | | | | | | | | SCORE | | | | | | | | | | | | | | | | | | | | | | | | | | | | | | | | | | | | | | | | | | | | | | | | | | | | | | | | RECORD SCORE HERE | | | | | | | 36 | | 34.1 |
|  |  |  |  |  |  |  |  | 0 | | | | | | | | | 1 | | | | | | | | | | | | 2 | | | | | | | | | | | | | | | | | | | | 3 | | | | | | | | 4 | | | | | | |  |  |  |  |  |  |  | 38 | | 34.6 |
| EDEMA | | | | | | | | Obvious edema of hands and feet pitting over tibia | | | | | | | | | No obvious edema of hands and feet pitting over tibia | | | | | | | | | | | | No edema | | | | | | | | | | | | | | | | | | | |  | | | | | | | |  | | | | | | |  | | | | | | | 40 | | 35.2 |
|  |  |  |  |  |  |  |  |  |  |  |  |  |  |  |  |  |  |  |  |  |  |  |  |  |  |  |  |  |  |  |  |  |  |  |  |  |  |  |  |  |  |  |  |  |  |  |  |  |  |  |  |  |  |  |  |  |  |  |  |  |  |  |  |  |  |  |  |  |  |  | 42 | | 35.7 |
| SKIN TEXTURE | | | | | | | | Very thin, gelatinous | | | | | | | | | Thin and smooth | | | | | | | | | | | | Smooth; medium thickness. Rash or superficial peeling | | | | | | | | | | | | | | | | | | | | Slight thickening. Superficial cracking & peeling, esp. hands, feet | | | | | | | | Thick and parchment like; superficial or deep cracking | | | | | | |  | | | | | | | 44 | | 36.2 |
|  |  |  |  |  |  |  |  |  |  |  |  |  |  |  |  |  |  |  |  |  |  |  |  |  |  |  |  |  |  |  |  |  |  |  |  |  |  |  |  |  |  |  |  |  |  |  |  |  |  |  |  |  |  |  |  |  |  |  |  |  |  |  |  |  |  |  |  |  |  |  | 46 | | 36.7 |
|  |  |  |  |  |  |  |  |  |  |  |  |  |  |  |  |  |  |  |  |  |  |  |  |  |  |  |  |  |  |  |  |  |  |  |  |  |  |  |  |  |  |  |  |  |  |  |  |  |  |  |  |  |  |  |  |  |  |  |  |  |  |  |  |  |  |  |  |  |  |  | 48 | | 37.3 |
| SKIN COLOUR Infant not crying | | | | | | | | Dark red | | | | | | | | | Uniformly pink | | | | | | | | | | | | Pale pink; variable over body | | | | | | | | | | | | | | | | | | | | Pale. Only pink over ears, lips, palms or soles | | | | | | | |  | | | | | | |  | | | | | | | 50 | | 37.8 |
|  |  |  |  |  |  |  |  |  |  |  |  |  |  |  |  |  |  |  |  |  |  |  |  |  |  |  |  |  |  |  |  |  |  |  |  |  |  |  |  |  |  |  |  |  |  |  |  |  |  |  |  |  |  |  |  |  |  |  |  |  |  |  |  |  |  |  |  |  |  |  | 52 | | 38.3 |
| SKIN OPACITY Trunk | | | | | | | | Numerous veins and enules clearly seen, esp over abdomen | | | | | | | | | Veins and tributaries seen | | | | | | | | | | | | a few large vessels clearly seen | | | | | | | | | | | | | | | | | | | A few large vessels seen indistinctly over the abdomen | | | | | | | | No blood vessels seen | | | | | | | |  | | | | | | | 54 | 38.9 | |
|  |  |  |  |  |  |  |  |  |  |  |  |  |  |  |  |  |  |  |  |  |  |  |  |  |  |  |  |  |  |  |  |  |  |  |  |  |  |  |  |  |  |  |  |  |  |  |  |  |  |  |  |  |  |  |  |  |  |  |  |  |  |  |  |  |  |  |  |  |  |  | 56 | 39.4 | |
|  |  |  |  |  |  |  |  |  |  |  |  |  |  |  |  |  |  |  |  |  |  |  |  |  |  |  |  |  |  |  |  |  |  |  |  |  |  |  |  |  |  |  |  |  |  |  |  |  |  |  |  |  |  |  |  |  |  |  |  |  |  |  |  |  |  |  |  |  |  |  | 58 | 39.9 | |
| LANUGO Over back | | | | | | | | No lanugo | | | | | | | | | Abundant; long and thick over whole back | | | | | | | | | | | | Hair thinning, esp. over lower back | | | | | | | | | | | | | | | | | | | Small amount of lanugo and bald areas | | | | | | | | At least half of the back devoid of lanugo | | | | | | | |  | | | | | | | 60 | 40.4 | |
|  |  |  |  |  |  |  |  |  |  |  |  |  |  |  |  |  |  |  |  |  |  |  |  |  |  |  |  |  |  |  |  |  |  |  |  |  |  |  |  |  |  |  |  |  |  |  |  |  |  |  |  |  |  |  |  |  |  |  |  |  |  |  |  |  |  |  |  |  |  |  | 62 | 41.0 | |
| PLANTAR CREASES | | | | | | | | No skin creases | | | | | | | | | Faint red marks over anterior half of sole | | | | | | | | | | | | Definite red marks over > anterior half; indentations over < anterior third | | | | | | | | | | | | | | | | | | | Indentations over more than anterior third | | | | | | | | Definite deep indentation over more than anterior third | | | | | | | |  | | | | | | | 64 | 41.5 | |
|  |  |  |  |  |  |  |  |  |  |  |  |  |  |  |  |  |  |  |  |  |  |  |  |  |  |  |  |  |  |  |  |  |  |  |  |  |  |  |  |  |  |  |  |  |  |  |  |  |  |  |  |  |  |  |  |  |  |  |  |  |  |  |  |  |  |  |  |  |  |  | 66 | 42.0 | |
|  |  |  |  |  |  |  |  |  |  |  |  |  |  |  |  |  |  |  |  |  |  |  |  |  |  |  |  |  |  |  |  |  |  |  |  |  |  |  |  |  |  |  |  |  |  |  |  |  |  |  |  |  |  |  |  |  |  |  |  |  |  |  |  |  |  |  |  |  |  |  | 68 | 42.6 | |
| NIPPLE FORMATION | | | | | | | | Nipple barely visible; no areola | | | | | | | | | Nipple well defined; areola smooth and flat; diam<0.75cm | | | | | | | | | | | | Areola stippled;, edge not raised; diameter <0.75cm | | | | | | | | | | | | | | | | | | | Areola stippled;, edge raised diameter >0.75cm | | | | | | | |  | | | | | | | |  | | | | | | |  |  | |
|  |  |  |  |  |  |  |  |  |  |  |  |  |  |  |  |  |  |  |  |  |  |  |  |  |  |  |  |  |  |  |  |  |  |  |  |  |  |  |  |  |  |  |  |  |  |  |  |  |  |  |  |  |  |  |  |  |  |  |  |  |  |  |  |  |  |  |  |  |  |  | **SCORE** |  | |
| BREAST SIZE | | | | | | | | No breast tissue palpable | | | | | | | | | Breast tissue on one or both sides <0.5cm diameter | | | | | | | | | | | | Breast tissue both sides; one or both 0.5-1.0 cm | | | | | | | | | | | | | | | | | | | Breast tissue both sides; one or both >1cm | | | | | | | |  | | | | | | | |  | | | | | | | Neurological |  | |
|  |  |  |  |  |  |  |  |  |  |  |  |  |  |  |  |  |  |  |  |  |  |  |  |  |  |  |  |  |  |  |  |  |  |  |  |  |  |  |  |  |  |  |  |  |  |  |  |  |  |  |  |  |  |  |  |  |  |  |  |  |  |  |  |  |  |  |  |  |  |  | Superficial |  | |
| EAR FORM | | | | | | | | Pinna flat and shapeless, or no incurving of edge | | | | | | | | | Incurving of part of edge of pinna | | | | | | | | | | | | Partial incurving of whole upper pinna | | | | | | | | | | | | | | | | | | | Well-defined incurving of whole of upper pinna | | | | | | | |  | | | | | | | |  | | | | | | | Total |  | |
|  |  |  |  |  |  |  |  |  |  |  |  |  |  |  |  |  |  |  |  |  |  |  |  |  |  |  |  |  |  |  |  |  |  |  |  |  |  |  |  |  |  |  |  |  |  |  |  |  |  |  |  |  |  |  |  |  |  |  |  |  |  |  |  |  |  |  |  |  |  |  |  |  |  |
| EAR FIRMNESS | | | | | | | | Pinna soft, easily folded, no recoil | | | | | | | | | Pinna soft, easily folded, slow recoil | | | | | | | | | | | | Cartilage to edge of pinna, but soft in places, ready recoil | | | | | | | | | | | | | | | | | | | Pinna firm, cartilage to edge, instant recoil | | | | | | | |  | | | | | | | |  | | | | | | | **GESTATIONAL AGE** | | |
|  |  |  |  |  |  |  |  |  |  |  |  |  |  |  |  |  |  |  |  |  |  |  |  |  |  |  |  |  |  |  |  |  |  |  |  |  |  |  |  |  |  |  |  |  |  |  |  |  |  |  |  |  |  |  |  |  |  |  |  |  |  |  |  |  |  |  |  |  |  |  | (weeks) |  | |
| GENITALIA MALE/FEMALE With hips half abducted | | | | | | | | Neither testis in scrotum. Labia majora widely separated, labia minora protruding | | | | | | | | | At least one testis high in scrotum. Labia majora almost cover labia minora | | | | | | | | | | | | At least one testis fully descended. Labia majora completely cover labia minora | | | | | | | | | | | | | | | | | | |  | | | | | | | |  | | | | | | | |  | | | | | | | By dates |  | |
|  |  |  |  |  |  |  |  |  |  |  |  |  |  |  |  |  |  |  |  |  |  |  |  |  |  |  |  |  |  |  |  |  |  |  |  |  |  |  |  |  |  |  |  |  |  |  |  |  |  |  |  |  |  |  |  |  |  |  |  |  |  |  |  |  |  |  |  |  |  |  | By U/S |  | |
|  |  |  |  |  |  |  |  |  |  |  |  |  |  |  |  |  |  |  |  |  |  |  |  |  |  |  |  |  |  |  |  |  |  |  |  |  |  |  |  |  |  |  |  |  |  |  |  |  |  |  |  |  |  |  |  |  |  |  |  |  |  |  |  |  |  |  |  |  |  |  | By score |  | |
|  |  |  |  |  |  |  |  |  |  |  |  |  |  |  |  |  |  |  |  |  |  |  |  |  |  |  |  |  |  |  |  |  |  |  |  |  |  |  |  |  |  |  |  |  |  |  |  |  |  |  |  |  |  |  |  |  |  |  |  |  |  |  |  |  |  |  |  |  |  |  | By Ballard |  | |
|  |  |  |  |  |  | |  | |  |  |  |  |  | |  |  | |  |  | |  | | |  | |  | |  | | |  | |  | | |  | | | |  | | |  | |  | **TOTAL SUPERFICIAL** | | | | | | | | | | | | | **SCORE** | | | | | | |  | | | | | |  | |

## Appendix 2: Major and Minor Birth Defects Recording

| **BIRTH DEFECTS** | | | | |  |  |  | |  |  |  |
| --- | --- | --- | --- | --- | --- | --- | --- | --- | --- | --- | --- |
| **TO BE FILLED BY THE DOCTOR ONLY** | | | | |  |  |  | |  |  |  |
| ** Please ask the caregiver kindly if you can examine the child. Find a private place.* | | | | | | | | | | | |
| *** Remember malformations tend to multiple. Please examine carefully.* | | | | | | | | | | | |
| **** Try your very best to arrange a photo - please include AP and lateral angle* | | | | | | | | | | | |
| Date of birth: | | / | / | 200___ |  | Birth weight: | | |  |  | grams |
| Date of examination: | | / | / | 200___ |  | EGA: | |  |  |  | weeks |
| Maximum fundal height on ANC card________cm Date maximum fundal height__________ | | | | | | | | | | | |
|  |  |  |  |  |  |  | |  |  |  |  |
| **In the following section circle any of the following that apply:** | | | | | | | |  |  |  |  |
| **ANC card** | growth: normal / slower than normal / faster than normal | | | | | | | |  |  |  |
| **General:** | cry - normal / abnormal….Please describe_________________________________ | | | | | | | | | | |
|  | hypertonic / hypotonic | | |  |  |  | |  |  |  |  |
| **Mouth** | Cleft palate / Cleft lip / protruding tongue / micrognathia / small arched palate | | | | | | | | | | |
| **Cranium** | Microcephalic / Hydrocephalic / Flat occiput (Down's) / Prominent occiput (Edward's) | | | | | | | | | | |
| **Eyes** | epicanthus / upward slanting / squint / micro-ophthalmia / anophthalmia / cyclopia | | | | | | | | | | |
| **Ears** | Low set / malformed / odd helices / deaf? | | | | |  | |  |  |  |  |
| **Nose** | Small / low nasal bridge | | |  |  |  | |  |  |  |  |
| **Neck** | Broad / short / | |  |  |  |  | |  |  |  |  |
| **Chest** | short sternum / hypoplastic nipples / widely spaced nipples | | | | | | | |  |  |  |
| **Hands** | amniotic banding / short hands and fingers / single palmar crease / polydactyly / | | | | | | | | | | |
|  | clenched with overlapping fingers / fused fingers (syndactyly) | | | | | | | |  |  |  |
| **Feet** | amniotic banding / extra toes / large gap between 1st and 2nd toe / small hallux / | | | | | | | | | | |
|  | rocker bottom feet / club foot | | | |  |  | |  |  |  |  |
| **Limbs** | bony aplasia / reduced limb length / agenesis / torsion / dislocated-clicky hips | | | | | | | | | | |
| **Gut** | diastasis recti / umbilical hernia / malformation of anus / omphalocele / gastroschisis | | | | | | | | | | |
|  | hepatomegaly / splenomegaly / scaphoid abdomen | | | | | | |  |  |  |  |
| **Skin** | skin tags / dry skin / haemangiomata / scalp defects / loose neck skin | | | | | | | | |  |  |
| **CVS** | Murmur Y/N - ASD / VSD / dextrocardia / PDA / absent peripheral pulses | | | | | | | | | |  |
| **Lungs** | Hypoplasia | |  |  |  |  | |  |  |  |  |
| **Genitalia** | Hypospadias / ambiguous / small labia / odd scrotum-testes x 2? / bladder exstrophy | | | | | | | | | | |
| **Spine** | Meningocoele (skin cover - no neural tissue) / meningomyelocoele (neural plate involved) | | | | | | | | | | |
|  | sacrococcygeal teratoma / kyphoscoliosis | | | | |  | |  |  |  |  |
| **Hair** | low set hairline / lanugo / hirsute | | | |  |  | |  |  |  |  |
| **Ask the mother:** | | 1. Did she have any abnormal children before? | | | | | | No / Yes….describe | | | |
|  |  | 2. Is her husband related to her- blood relative? | | | | | | No / Yes….describe | | | |
|  |  | 3. Did the mother take any drugs not on the ANC card? | | | | | | | No / Yes…list | | |
|  |  | the drug name and amount and the approximate gestation | | | | | | | |  |  |
| **Other details *(Use more paper if necessary)*:** | | | | |  |  | |  |  |  |  |

1. World Health Organization. *Roll Back Malaria Global Partnership*, (<http://rbm.who.int/newdesign2/gp/gp.htm>) [↑](#endnote-ref-2)
2. Shulman CE, Dorman EK. Reducing childhood mortality in poor countries - Importance and prevention of malaria in pregnancy. *Transactions of the Royal Society of Tropical Medicine and Hygiene* 2003;97(1):30-5. [↑](#endnote-ref-3)
3. McGregor IA, Wilson ME, Billewicz WZ. Malaria infection of the placenta in The Gambia, West Africa; its incidence and relationship to stillbirth, birthweight and placental weight. *Trans.R.Soc.Trop.Med.Hyg*. 1983;77(2):232-44. [↑](#endnote-ref-4)
4. Brabin BJ. An analysis of malaria in pregnancy in Africa. *Bull.World Health Organ* 1983;61(6):1005-16. [↑](#endnote-ref-5)
5. Nosten F, McGready R, Simpson JA, Thwai KL, Balkan S, Cho T et al. Effects of Plasmodium vivax malaria in pregnancy. *Lancet* 1999;354(9178):546-9. [↑](#endnote-ref-6)
6. Steketee RW, Wirima JJ, Hightower AW, Slutsker L, Heymann DL, Breman JG. The effect of malaria and malaria prevention in pregnancy on offspring birthweight, prematurity, and intrauterine growth retardation in rural Malawi. *Am J Trop Med Hyg* 1996; 55(1 Suppl):33-41. [↑](#endnote-ref-7)
7. Van geertruyden JP; Thomas F, Erhart A, D’Alessandro U. The contribution of malaria in pregnancy to perinatal mortality. *Am J Trop Med Hyg* 2004 71(Suppl 2), 35-40. [↑](#endnote-ref-8)
8. Bruce-Chwatt LJ. *Malaria in African infants and children in Southern Nigeria*. 173-200. 1952. [↑](#endnote-ref-9)
9. Jelliffe EFP. Low birth weight and malarial infection of the placenta. *Bulletin of the World Health Organization* 38, 69-78. 1968. [↑](#endnote-ref-10)
10. Kortmann HF. *Malaria and pregnancy. MD Thesis* 1972. Utrecht: Drukkerijk Elinkwijk. 1972. [↑](#endnote-ref-11)
11. Newman RD, Parise ME, Slutsker L, Nahlen B, Steketee RW. Safety, efficacy and determinants of effectiveness of antimalarial drugs during pregnancy: implications for prevention programmes in Plasmodium falciparum-endemic sub-Saharan Africa. *Tropical Medicine & International Health* 2003;8(6):488-506. [↑](#endnote-ref-12)
12. Kramer MS. Determinants of low birth weight: methodological assessment and meta-analysis. *Bulletin-of-the-World-Health-Organization* 65(5), 663-737. 1987. [↑](#endnote-ref-13)
13. Garner P, Brabin B. A review of randomized controlled trials of routine antimalarial drug prophylaxis during pregnancy in endemic malarious areas. *Bulletin of the World Health Organization* 1994; 72(1):89-99. [↑](#endnote-ref-14)
14. McCormick M-C. The contribution of low birth weight to infant mortality and childhood morbidity. New-England-journal-of-medicine,-*The. N-Engl-J-Med* 1985;312(2):82-90. [↑](#endnote-ref-15)
15. Murphy SC, Breman JG. Gaps in the childhood malaria burden in Africa: Cerebral malaria, neurological sequelae, anemia, respiratory distress, hypoglycemia, and complications of pregnancy. *American Journal of Tropical Medicine and Hygiene* 2001;64(1-2):57-67. [↑](#endnote-ref-16)
16. Steketee RW, Nahlen BL, Parise ME, Menendez C. The burden of malaria in pregnancy in malaria-endemic areas. *American Journal of Tropical Medicine and Hygiene* 2001;64(1-2):28-35. [↑](#endnote-ref-17)
17. Mc Gregor IA, Wilson ME, Billewicz WZ, 1983. Malaria infection of the placenta in the Gambia, West Africa: Its incidence and relationship to stillbirth, birthweight and placental weight.  *Trans R Soc Trop Med Hyg* 77: 232-244 [↑](#endnote-ref-18)
18. Ter Kuile FO, Terlouw DJ, Phillips-Howard PA, Hawley WA, Friedman JF, Kariuki SK, Shi YP, Kolczak MS, Lal AA, Vulule JM, Nahlen BL (2003). Reduction of malaria during pregnancy by permethrin-treated bed nets in an areas of intense perennial malaria transmission in Western Kenya. *American Journal of Tropical Medicine and Hygiene* 68 (Suppl. 4), 50-60. [↑](#endnote-ref-19)
19. Garner P, Brabin B. A review of randomized controlled trials of routine antimalarial drug prophylaxis during pregnancy in endemic malarious areas. *Bulletin of the World Health Organization* 1994; 72(1):89-99. [↑](#endnote-ref-20)
20. Kramer MS, 1987. Determinants of low birth weight: Methodological assessment and meta-analysis. *Bull World Health Organization65: 663-737*  [↑](#endnote-ref-21)
21. Steketee RW, Wirma JJ, Bloland PB et al 1996. Impairment of a pregnant woman’s acquired ability to limit P.fal by infection with HIV-1 *Am j Trop Med Hy 55 (suppl): 42-49.* [↑](#endnote-ref-22)
22. Allen S, Van de perre P, Serufilira A, Lepage P, Carael M, DeClercq A, Tice J, Black D, Nsengumuremyi F, Ziegler J, et al. Human immunodeficiency virus and malaria in a representative sample of childbearing women in Kigali, Rwanda. *J Infect Dis. 1991 Jul;164(1):67-71*. [↑](#endnote-ref-23)
23. Steketee RW, Wirima JJ, Bloland PB, Chilima B, Mermin JH, Chitsulo L, Breman JG. Impairment of a pregnant woman's acquired ability to limit *Plasmodium falciparum* by infection with human immunodeficiency virus type-1.*Am J Trop Med Hyg. 1996;55(1 Suppl):42-9.* [↑](#endnote-ref-24)
24. Verhoeff FH, Brabin BJ, Hart CA, Chimsuku L, Kazembe P, Broadhead RL. Increased prevalence of malaria in HIV-infected pregnant women and its implications for malaria control. *Trop Med Int Health. 1999 Jan;4(1):5-12*. [↑](#endnote-ref-25)
25. van Eijk AM, Ayisi JG, ter Kuile FO, Misore AO, Otieno JA, Rosen DH, Kager PA, Steketee RW, Nahlen BL. Risk factors for malaria in pregnancy in an urban and peri-urban population in western Kenya. *Trans R Soc Trop Med Hyg. 2002 Nov-Dec;96(6):586-92.*  [↑](#endnote-ref-26)
26. ter Kuile FO, Parise ME, Verhoeff FH, Udhayakumar V, Newman RD, van Eijk AM, Rogerson SJ, Steketee RW. The burden of co-infection with human immunodeficiency virus type 1 and malaria in pregnant women in sub-saharan Africa. *Am J Trop Med Hyg*. 2004 Aug;71(2 Suppl):41-54. Review. [↑](#endnote-ref-27)
27. Ayisi JG, van Eijk AM, ter Kuile FO, Kolczak MS, Otieno JA, Misore AO, Kager PA, Steketee RW, Nahlen BL. The effect of dual infection with HIV and malaria on pregnancy outcome in western Kenya. *AIDS. 2003 Mar 7;17(4):585-94.*  [↑](#endnote-ref-28)
28. Ticconi C, Mapfumo M, Dorrucci M, Naha N, Tarira E, Pietropolli A, Rezza G. Effect of maternal HIV and malaria infection on pregnancy and perinatal outcome in Zimbabwe. *J Acquir Immune Defic Syndr. 2003 Nov 1;34(3):289-94*. [↑](#endnote-ref-29)
29. Bloland PB, Wirima JJ, Steketee RW, Chilima B, Hightower A, Breman JG. Maternal HIV infection and infant mortality in Malawi: evidence for increased mortality due to placental malaria infection. *AIDS. 1995 Jul;9(7):721-6.*  [↑](#endnote-ref-30)
30. Parise ME, Ayisi JG, Nahlen BL, Schultz LJ, Roberts JM, Misore A et al. Efficacy of sulfadoxine-pyrimethamine for prevention of placental malaria in an area of Kenya with high prevalence of malaria and human immunodeficiency virus infection. *American Journal of Tropical Medicine & Hygiene 1998; 59:813-822*. [↑](#endnote-ref-31)
31. Verhoeff ea. An evaluation of the effects of intermittent sulfadoxine-pyrimethamine treatment in pregnancy on parasite clearance and risk of low birth weight in rural Malawi. *Ann Trop Med Parasitol 1998;92.* [↑](#endnote-ref-32)
32. Annual Health Statistical *Bulletin-CBoH, 2003*, Zambia [↑](#endnote-ref-33)
33. ? National Malaria Situation Analysis –*NMCC, Zambia*. [↑](#endnote-ref-34)
34. WHO World Health Statistics 2006 <http://www.who.int/whosis/en/> - *Country Health System Fact Sheet 2006 (Zambia).* [↑](#endnote-ref-35)
35. ? [Spencer DC.](http://www.ncbi.nlm.nih.gov/pubmed/17884000?ordinalpos=1&itool=EntrezSystem2.PEntrez.Pubmed.Pubmed_ResultsPanel.Pubmed_RVDocSum) Preventing bacterial disease in the HIV-infected of sub-Saharan Africa: the role of cotrimoxazole and the pneumococcal vaccines. Curr HIV/AIDS Rep. 2007 Aug;4(3):141-6. Review. [↑](#endnote-ref-36)
36. Chintu c, Bhat GJ, Walker AS, Mulenga V, Sinyinza F, Lishimpi K, Farrelly L,Kaganson N, Zumla A, Gillespie SH, Nunn AJ,Gibb DM, 2004. Cotrimoxazole as prophylaxis against opportunistic infections in HIV-infected Zambian children (CHAP): a double-blind randomised placebo-controlled trial. *Lancet 364*: 1865-1871 [↑](#endnote-ref-37)
37. ? Hauth JC, Goldenberg RL, Andrew WN, Dubard MB, Copper RL. Reduced incidence of preterm delivery with metronidazole and erythromycin in women with bacterial vaginosis.NEngl. J. Med 1995 Dec 28, 333 (26); 1732-6 [↑](#endnote-ref-38)
38. ? Walter J, Mwiya M, Scott N, Kasonde P, Sinkala M, Kankasa C, Kauchali S, Aldrovandi GM, Thea DM, Kuhn L. Reduction in preterm delivery and neonatal mortality after the introduction of antenatal cotrimoxazole prophylaxis among HIV-infected women with low CD4 cell counts. *J Infect Dis* 2006;194:1510-1518 [↑](#endnote-ref-39)
39. Forna F, McConnell M, Kitabire FN, et al. Systematic review of the safety of trimethoprim-sulfamethoxazole for prophylaxis in HIV-infected pregnant women: implications for resource-limited settings. AIDS Rev **2006**; 8:24–36. [↑](#endnote-ref-40)
40. *World Health Organization, 2006*. Guidelines on co-trimoxazole prophylaxis for HIV-related infections among children, adolescents and adults : recommendations for a public health approach [↑](#endnote-ref-41)
41. Omar S, Bakari A, Owiti A, Adagu I, Warhurst D. Co-trimoxazole compared with SP in the treatment of uncomplicated malaria in Kenyan children. *Trans R Soc trop Med Hy 2001; 95: 657-60* [↑](#endnote-ref-42)
42. Anglaret X, Chene G, Attia A, et al. Early chemoprophylaxis with trimethoprim-sulphamethoxazole for HIV-1-infected adults in Abidjan, Cote d’Ivoire: a randomized tria. *Lancet 1999: 353: 1463-68* [↑](#endnote-ref-43)
43. ? Mermin J, Lule J, EkwaruJ, et al. Effect of co-trimoxazole prophylaxis on morbidity, mortality, CD4-cell count and viral load in HIV-infection in rural Uganda. *Lancet 2004: 364: 1428-43* [↑](#endnote-ref-44)
44. ? Brentlinger PE, Behrens CB, Micek MA. Challenges in the concurrent management of malaria and HIV in pregnancy in sub-Saharan Africa. *Lancet Infect Dis* 2006;6:100-111 [↑](#endnote-ref-45)
45. Janossy G, Jani I, Gohde W. Affordable CD4(+) T-cell counts on 'single-platform' flow cytometers I. Primary CD4 gating. *Br J Haematol* 2000 Dec; 111(4):1198-208. [↑](#endnote-ref-46)
46. Trape JF. Rapid evaluation of malaria parasite density and standardization of thick smear examination for epidemiological investigations. *Transactions of the Royal Society of Tropical Medicine & Hygiene 1985; 79(2):181-184.* [↑](#endnote-ref-47)
47. ? Kaplan JE, Masur H, Holmes KK: Guidelines for preventing opportunistic infections among HIV-infected persons--2002. Recommendations of the U.S. Public Health Service and the Infectious Diseases Society of America. *MMWR Recomm Rep* 2002, 51:1-52. [↑](#endnote-ref-48)
48. Dubowitz LMS, Dubowitz V, Goldberg C. Clinical assessment of gestational age in the newborn infant. *Journal of Pediatrics 1970; 77:1-10*. [↑](#endnote-ref-49)
49. Guay LA, Musoke P, Fleming T, Bagenda D, Allen M, Nakabiito C et al. Intrapartum and neonatal single-dose nevirapine compared with zidovudine for prevention of mother-to-child transmission of HIV-1 in Kampala, Uganda: HIVNET 012 randomised trial. *Lancet 1999; 354:795-802* [↑](#endnote-ref-50)
50. Bulmer JN, Rasheed FN, Francis N, Morrison L, Greenwood BM. Placental malaria. I. Pathological classification. *Histopathology 1993; 22:211-218*. [↑](#endnote-ref-51)
51. Walter PR, Garin Y, Blot P. Placental pathologic changes in malaria: a histologic and ultrastructural study. *American Journal of Pathology 1982; 109:330-342.* [↑](#endnote-ref-52)
52. Peters PJ, Thigpen MC, Parise ME, Newman RD.Safety and toxicity of sulfadoxine/pyrimethamine: implications for malaria prevention in pregnancy using intermittent preventive treatment. Drug Saf. 2007;30(6):481-501. [↑](#endnote-ref-53)
